# Supplementary material for: Transcriptomics of Plasmodium vivax rosetting
Source: Mem Inst Oswaldo Cruz. 2026 Mar 9;121:e250198. doi: 10.1590/0074-02760250198 (PMC12974587; doi:10.1590/0074-02760250198)
Supplement: Supplementary material [file 1678-8060-mioc-121-e250198-s1.pdf]

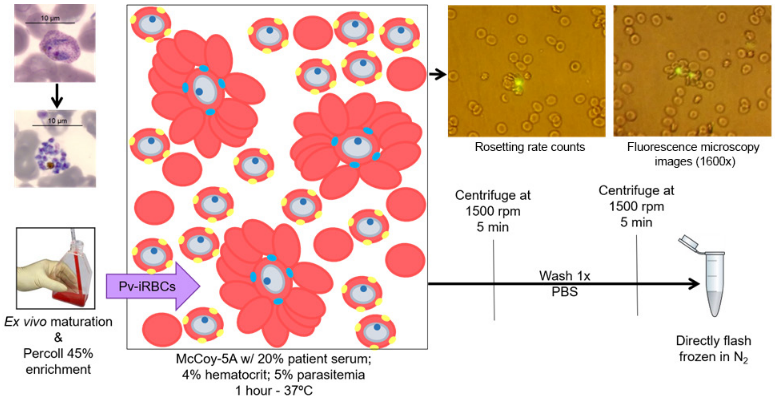

Fig. 1: rosetting rates assessment. Rosetting methods scheme to determine the rosetting capacity of distinct populations of *Plasmodium vivax*-infected red blood cells (*Pv*-iRBCs) from *vivax* malaria patient isolates.

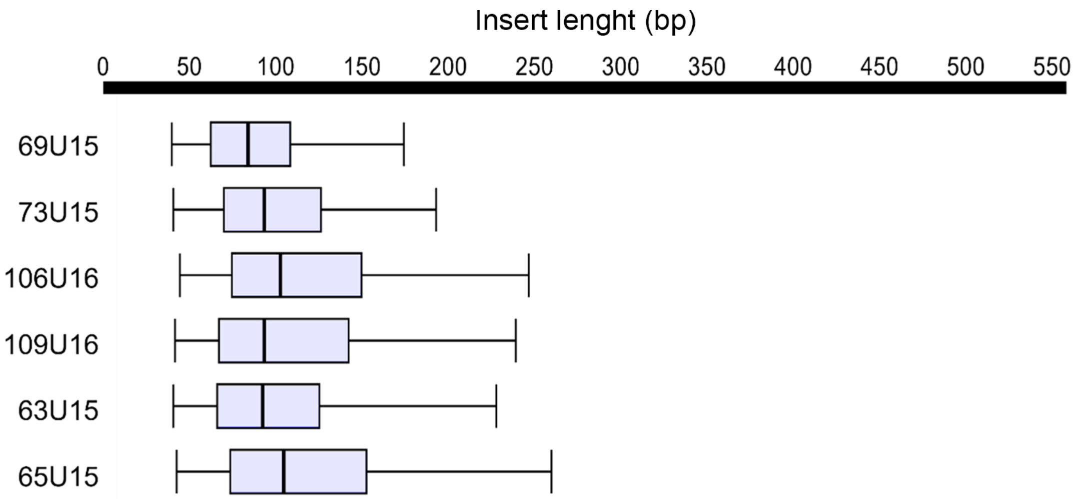

Fig. 2: boxplot summarising the insert length distribution of paired-end reads of all RNA sequenced libraries. *bp: base pair.

TABLE I

Patient profiles and sample details of *Plasmodium vivax* clinical isolates evaluated on rosetting assays

Patient code	Sex	Age	Parasites/μL	WBC (x10^3/μl)	RBC (x10^6/μL)	HGB (g/dL)	HCT (%)	PLT (x10^3/μL)	LYM (x10^3/μL)	NEUT (x10^3/μL)	Rosetting rates (%)	Group analysis
**59U15**	M	41	17920	5.9	5.07	14.1	45.8	43	0.6	4.8	56.88	HR
**61U15**	F	42	7400	3.8	4.66	13	42.4	132	1.1	2	44.49	HR
**62U15**	F	32	13400	5.5	4.04	11	34.6	42	1.2	3.8	9.70	LR
**63U15**	F	36	16000	3.6	4.82	12.7	41.7	79	1.1	1.9	7.00	LR
**65U15**	M	49	17120	4.7	4.6	13	41.7	95	1.9	2.7	7.00	LR
**68U15**	M	ND	10000	6.2	4.13	11.3	36.6	81	0.7	5.2	8.76	LR
**69U15**	M	56	17200	3	4.39	13.3	42.2	43	0.3	2.6	24.40	HR
**70U15**	F	25	5400	2.3	5.49	15.5	49.8	67	0.6	1.5	12.00	HR
**73U15**	M	23	ND	ND	ND	ND	ND	26.3	3.0	0.4	5.60	LR
**76U15**	M	24	10800	ND	ND	ND	ND	24.3	3.1	1.3	5.86	LR
**78U15**	M	26	13040	6.7	4.58	11.5	37.9	76	1.2	5.2	13.02	HR
**87U15**	M	29	8800	4	5.48	15.7	48.3	36	0.5	2.5	9.09	LR
**94U15**	F	43	9200	4.8	3.87	10.6	32.9	11	1	3.7	4.03	LR
**95U15**	M	59	4800	5	5.08	13.9	42.6	83	0.9	3.9	4.84	LR
**106U16**	M	57	9200	7.3	5.09	14.2	46.3	94	0.7	6	30.00	HR
**109U16**	M	50	19200	1.3	4.64	11.7	39.7	47	0.3	1	6.00	LR

M: male; F: female; WBC: white blood cells; RBC: red blood cells; HGB: haemoglobin; HCT: haematocrit; PLT: platelets; LYM: lymphocytes; NEUT: neutrophils; LR: low rosetting; HR: high rosetting; ND: not determined. Samples shaded in light grey were RNA sequenced.

TABLE II

Bioanalyzer measurements after RNA extraction with RNeasy Micro kit of rosetting samples (*Plasmodium vivax*-infected red blood cells: *Pv*-iRBC); and control (*Plasmodium falciparum* FCR3 S1.2-infected red blood cells: *Pf*-iRBC)

Isolate code	*Pv*-iRBC sample code	RNA [pg/μL]	RIN
63U15	15	8.914	9.8
65U15	16	21.499	9.7
69U15	11	13.529	6.6
73U15	12	4.925	7.4
106U16	13	17.234	6.3
109U16	14	1.87	N/A
Pf FCR3 S1.2	17	3.264	N/A

RIN: RNA integrity number; N/A: undetermined. *P. falciparum* FCR3 S1.2 (~10^6 *Pf*-iRBC; shaded in light grey) sample was used as highly rosetting (> 80%) control strain.

TABLE III

Quality and quantity controls after cDNA library generation and amplification of rosetting in samples (*Plasmodium vivax*-infected red blood cells: *Pv*-iRBC); and control (*Plasmodium falciparum* FCR3 S1.2-infected red blood cells: *Pf*-iRBC)

Isolate code	*Pv*-iRBC sample code	cDNA [pg/µL]	cDNA [ng/µL]	V to 20 ng (µL)	Total cDNA [ng/75µL]	V_T to 20 ng per reaction from 75 µL after shearing (µL)	V Elution Buffer (µL)	Total cDNA [ng/reaction]	Nº of PCR cycles for Low Input Library amplification	Fragment average size (bp)	Molarity [pmol/L]
63U15	15	124671	124.671	0.16	9350.300	1.2	8.8	20	6	366	1.97E+05
65U15	16	53184.4	53.184	0.38	3988.827	2.8	7.2	20	6	416	1.51E+05
69U15	11	137982	137.982	0.14	10348.684	1.1	8.9	20	6	396	1.85E+05
73U15	12	249417	249.417	0.08	18706.261	0.6	9.4	20	6	310	5.69E+04
106U16	13	10061.3	10.061	1.99	754.595	12.0	-	1.609	6	369	7.98E+05
109U16	14	413707	413.707	0.05	31028.009	0.4	9.6	20	6	393	8.56E+04
Pf FCR3 S1.2	17	246122.25	246.122	0.08	18459.169	0.6	9.4	20	6	378	8.87E+04

V: volume; V_T: total volume; bp: base pairs. *P. falciparum* FCR3 S1.2 (~10^6 *Pf*-iRBC; shaded in light grey) sample was used as highly rosetting (>80%) control strain.

TABLE IV

Library quantification by qPCR and sample pool for Illumina HiSeq 2500 load and run of rosetting samples (*Plasmodium vivax*-infected red blood cells: *Pv*-iRBC); and control (*Plasmodium falciparum* FCR3 S1.2-infected red blood cells: *Pf*-iRBC)

Sample code	*Pv*-iRBC sample code	Dilution	Cq mean	Cq error	Mean [pM]	Error [pM]	Total [pM]	Total [nM]	*447bp from DNA fragments	Library concentration [nM]		V at dilution 2 nM (µL)	V ddH$_2$O (µL)	V_T per sample (µL)
63U15	15	1.00E-09	13.39	0.58	8.79E-01	2.97E-01	8.79E+08	878500.00	3.93E+11	1072922	dil -5	10.729221	1.30	5.70
65U15	16	1.00E-09	13.83	0.77	6.72E-01	3.24E-01	6.72E+08	672100.00	3.00E+11	722184.4	dil -5	7.2218438	1.94	5.06
69U15	11	1.00E-09	15.13	0.75	2.73E-01	1.74E-01	2.73E+08	272800.00	1.22E+11	307933.3	dil -5	3.0793333	4.55	2.45
73U15	12	1.00E-09	12.55	1.40	2.09E+00	1.88E+00	2.09E+09	2090000.00	9.34E+11	3013645	dil -6	3.0136452	4.65	2.35
106U16	13	1.00E-10	17.23	0.23	5.40E-02	8.57E-03	5.40E+08	540000.00	2.41E+11	654146.3	dil -5	6.5414634	2.14	4.86
109U16	14	1.00E-09	14.44	0.33	4.03E-01	9.00E-02	4.03E+08	403000.00	1.80E+11	458374	dil -5	4.5837405	3.05	3.95
Pf FCR3 S1.2	17	1.00E-09	18.12	2.45	7.05E-02	8.30E-02	7.05E+07	70450.00	3.15E+10	83309.9	dil -4	8.330882	1.68	5.32

V: volume; V_T/sample = 7.0µL; bp: base pairs. *P. falciparum* FCR3 S1.2 (~10^6 *Pf*-iRBC; shaded in light grey) sample was used as highly rosetting (>80%) control.

TABLE V

Raw data (reads) description and statistics output from RNA-sequencing after performing quality control and trimming of rosetting samples

Isolate code	RNA-seq ID	Lane	Index	Filename	Total sequences (FastQ)	Sequence length	%GC	Total nº of reads after trimming (%) (Trimmomatic)
69U15	11	2	CAGATC	11_S18_L001_R1_001.fastq.gz<br>11_S18_L001_R2_001.fastq.gz	444674444674	101<br>101	46<br>47	436832<br>(93.8%)
73U15	12	2	ACTTGA	12_S19_L001_R1_001.fastq.gz<br>12_S19_L001_R2_001.fastq.gz	465530<br>465530	101<br>101	45<br>46	457871<br>(98,4%)
106U16	13	2	ACAGTG	13_S16_L001_R1_001.fastq.gz<br>13_S16_L001_R2_001.fastq.gz	741000<br>741000	101<br>101	49<br>49	726393<br>(98.0%)
109U16	14	2	GATCAG	14_S20_L001_R1_001.fastq.gz<br>14_S20_L001_R2_001.fastq.gz	247006<br>247006	101<br>101	46<br>46	244626<br>(99.0%)
63U15	15	2	TAGCTT	15_S21_L001_R1_001.fastq.gz<br>15_S21_L001_R2_001.fastq.gz	556333<br>556333	101<br>101	47<br>47	550489<br>(98.9%)
65U15	16	2	GGCTAC	16_S23_L001_R1_001.fastq.gz<br>16_S23_L001_R2_001.fastq.gz	553823<br>553823	101<br>101	45<br>46	222254<br>(99.5%)
Pf FCR3<br>S1.2	17	2	CTTGTA	17_S23_L001_R1_001.fastq.gz<br>17_S23_L001_R2_001.fastq.gz	223390<br>223390	101<br>101	29<br>30	218478<br>218478<br>(97.8%)

Quality control and trimming of the raw data (paired-end reads) was performed using FastQC and Trimmomatic as recommended (see methods section). All samples passed FastQC quality controls with none of the sequences flagged as poor quality. The sample reference used for all field isolates was *Plasmodium vivax* P01 reference genome[37] for downstream analysis. The sample reference used for *Plasmodium falciparum* FCR3 S1.2 (~10^6 *Pf*-RBC; shaded in light grey) control sample was *P. falciparum* IT reference genome for downstream analysis.

TABLE VI

Alignment and mapping summary report for Pv-iRBC rosetting samples RNA sequenced

	Isolate code (RNA-seq ID)						*P. falciparum* FCR3 S1.2
	69U15 (11)	73U15 (12)	106U16 (13)	109U16 (14)	63U15 (15)	65U15 (16)	
Left reads							
Input	436832	457871	726393	244626	550489	218478	537931
Mapped (% of input)	220515 (50.5%)	268217 (58.6%)	241490 (33.2%)	153683 (62.8%)	331462 (60.2%)	127874 (58.5%)	299290 (55.6%)
Multiple alignments (% of mapped)	4044 (1.8%)	4963 (1.9%)	4997 (2.1%)	2479 (1.6%)	6071 (1.8%)	3103 (2.4%)	33175 (11.1%)
Multiple alignments (>20)	27	30	55	17	32	20	237
Right reads							
Input	436832	457871	726393	244626	550489	218478	537931
Mapped	219709 (50.3%)	267472 (58.4%)	241068 (33.2%)	153046 (62.6%)	330121 (60.0%)	127278 (58.3%)	301231 (56.0%)
Multiple alignments	4561 (2.1%)	5608 (2.1%)	6237 (2.6%)	2776 (1.8%)	6813 (2.1%)	3376 (2.7%)	35211 (11.7%)
Multiple alignments (>20)	27	30	57	17	31	20	239
Overall read mapping rate	50.40%	58.50%	33.20%	62.70%	60.10%	58.40%	55.80%
Aligned pairs	198574	241964	210760	139372	301369	114319	270875
Multiple alignments (% of aligned pairs)	2864 (1.4%)	3680 (1.5%)	2816 (1.3%)	1955 (1.4%)	4825 (1.6%)	2259 (2.0%)	27722 (10.2%)
Discordant alignments (% of aligned pairs)	3479 (1.8%)	3272 (1.4%)	3360 (1.6%)	2646 (1.9%)	4114 (1.4%)	1374 (1.2%)	7248 (2.7%)
Concordant pair alignment rate	44.70%	52.10%	28.60%	55.90%	54.00%	51.70%	49.00%
Coverage	0.135	1.640	1.429	0.945	2.043	0.775	1.836
RPKM	12.420	15.175	13.750	8.951	19.699	7.533	80.939

Data table summarising the alignment and mapping results against the *Plasmodium vivax* P01 reference genome obtained using TopHat2 on our raw data (reads) for the clinical isolates that were sequenced. Both pair of reads (left and right) were mapped, checked for multiple alignments and concordance of pair alignment. Same analysis is shown for our control sample of *Plasmodium falciparum* S20 against the *P. falciparum* IT reference genome. The estimation of sequencing coverage was calculated using the Lander/Waterman equation $C = 2LN/G$, where C stands for coverage, 2*L is paired-end[70] end sequencing multiplied by L, the read length (100 bp), N is the number of reads aligning to genes (features) and G is the haploid genome length, 29.05 Mbp for *P. vivax* P01 and 23.18 Mbp for *P. falciparum* IT reference genomes (https://www.illumina.com/documents/products/technotes/technote_coverage_calculation.pdf). RPKM (reads per kilo base per million mapped reads) was calculated using the following equation: $RPKM = (C * 10^3 * 10^6) / (N * L)$, where C stands for the number of reads per sample, N is the total number of reads obtained in the experiment and L is the total exon length (in bp), 12,898,530 bp for *P. vivax* P01 and 12,355,032 bp for *P. falciparum* IT reference genomes.

TABLE VII

Htseq-count read alignment and feature count summary report for *Plasmodium vivax* field isolates RNA sequenced and *Plasmodium falciparum* FCR3 S1.2 control

Sample code	63U15	65U15	69U15	73U15	106U16	109U16	*P. falciparum* FCR3 S1.2
Reads alignment	15	16	11	12	13	14	
genes (features)	107755	44867	75519	92522	88208	51002	149687
ambiguous	42310	14469	32979	38631	34115	21229	12982
not unique	38046	21968	33072	37313	53285	16702	228897

Data table summarizing the read count to genes against *P. vivax* P01 reference genome obtained using htseq-count on our aligned and mapped data (reads) for the clinical isolates that were sequenced. For all samples only good quality alignment were considered. Same analysis is shown for *P. falciparum* FCR3 S1.2 against the *P. falciparum* IT reference genome.

TABLE VIII

List of differentially expressed genes between samples of the *Plasmodium vivax*-infected red blood cells (*Pv*-iRBC) showing high versus low rosetting rates comparison group

Exon ID	TL	Reads count	CVGE	RPKM	Mean counts	Log2FC	SD	Wald stat	p-value	q-value	Gene ID	Symbol	Product description
exon_PVP01_0616000-E1	711	913	128.411	22078.487	163.285	3.304	0.511	6.468	9.94E-11	2.20E-07	PVP01_0616000	P28	ookinete surface protein P28, putative
exon_PVP01_1465500-E1	888	652	73.423	12624.168	115.581	3.835	0.587	6.535	6.37E-11	2.20E-07	PVP01_1465500		conserved Plasmodium protein, unknown function
exon_PVP01_1251100-E1	4824	307	6.364	1094.206	55.445	3.790	0.593	6.387	1.69E-10	2.50E-07	PVP01_1251100	CCp2	LCCL domain-containing protein
exon_PVP01_0526400-E1	960	797	83.021	14274.313	141.884	3.620	0.593	6.105	1.03E-09	1.14E-06	PVP01_0526400		conserved Plasmodium protein, unknown function
exon_PVP01_0616100-E1	660	2132	323.030	55540.706	376.739	3.269	0.544	6.006	1.91E-09	1.69E-06	PVP01_0616100	P25	ookinete surface protein P25
exon_PVP01_1251100-E2	4824	1264	26.202	4505.136	225.585	3.273	0.563	5.811	6.22E-09	3.67E-06	PVP01_1251100		LCCL domain-containing protein
exon_PVP01_1344600-E1	399	473	118.546	20382.450	86.962	3.586	0.618	5.800	6.64E-09	3.67E-06	PVP01_1344600		conserved Plasmodium protein, unknown function
exon_PVP01_1345600-E1	1077	740	68.709	11813.651	132.486	3.456	0.596	5.802	6.55E-09	3.67E-06	PVP01_1345600		conserved Plasmodium protein, unknown function
exon_PVP01_0530400-E1	225	177	78.667	13525.673	31.390	3.446	0.615	5.603	2.11E-08	1.04E-05	PVP01_0530400		conserved Plasmodium protein, unknown function
exon_PVP01_0822100-E1	651	705	108.295	18619.854	124.993	3.240	0.581	5.581	2.39E-08	1.06E-05	PVP01_0822100		conserved Plasmodium protein, unknown function
exon_PVP01_1318600-E1	807	760	94.176	16192.287	138.388	3.253	0.590	5.509	3.61E-08	1.46E-05	PVP01_1318600	PIP3	PhIL1 interacting protein PIP3, putative
exon_PVP01_1140300-E2	1368	758	55.409	9526.892	134.673	2.937	0.539	5.449	5.07E-08	1.74E-05	PVP01_1140300		conserved Plasmodium protein, unknown function
exon_PVP01_1452800-E2	3297	222	6.733	1157.716	39.075	2.990	0.549	5.448	5.09E-08	1.74E-05	PVP01_1452800	ULG8	upregulated in late gametocytes ULG8, putative
exon_PVP01_1229400-E2	1005	883	87.861	15106.462	162.515	3.455	0.642	5.379	7.47E-08	2.36E-05	PVP01_1229400		lactate dehydrogenase, putative
exon_PVP01_0106900-E1	2628	226	8.600	1478.602	40.996	3.327	0.624	5.337	9.48E-08	2.74E-05	PVP01_0106900	MSH2-2	DNA mismatch repair protein MSH2, putative
exon_PVP01_1467500-E1	1950	1122	57.538	9892.963	198.215	2.755	0.517	5.328	9.91E-08	2.74E-05	PVP01_1467500		conserved Plasmodium protein, unknown function
exon_PVP01_0508600-E1	897	661	73.690	12670.016	116.349	3.014	0.580	5.199	2.00E-07	5.22E-05	PVP01_0508600	CryPH	crystalloid-specific PH domain-containing protein, putative
exon_PVP01_1255400-E2	2625	496	18.895	3248.782	88.802	3.061	0.591	5.182	2.19E-07	5.39E-05	PVP01_1255400	LAP5	LCCL domain-containing protein, putative
exon_PVP01_0526400-E2	960	301	31.354	5390.926	53.367	3.335	0.651	5.123	3.01E-07	7.02E-05	PVP01_0526400		conserved Plasmodium protein, unknown function
exon_PVP01_0914700-E1	1728	402	23.264	3999.912	74.361	3.231	0.639	5.061	4.18E-07	8.45E-05	PVP01_0914700	MAPK2	mitogen-activated protein kinase 2, putative
exon_PVP01_1240200-E1	822	131	15.937	2740.108	23.976	3.398	0.672	5.060	4.19E-07	8.45E-05	PVP01_1240200		enkurin domain-containing protein, putative
exon_PVP01_1412100-E1	1488	4075	273.858	47086.110	753.155	3.277	0.647	5.065	4.07E-07	8.45E-05	PVP01_1412100		meiosis-specific nuclear structural protein 1, putative
exon_PVP01_1252100-E1	459	164	35.730	6143.266	28.937	3.080	0.611	5.045	4.55E-07	8.76E-05	PVP01_1252100		conserved protein, unknown function
exon_PVP01_0530800-E3	1353	5277	390.022	67059.056	953.815	2.815	0.565	4.978	6.42E-07	1.16E-04	PVP01_0530800		alpha tubulin 2, putative
exon_PVP01_1136600-E1	366	421	115.027	19777.398	73.402	2.850	0.573	4.975	6.53E-07	1.16E-04	PVP01_1136600		conserved Plasmodium protein, unknown function
exon_PVP01_1229400-E3	1005	1860	185.075	31821.087	340.334	3.150	0.635	4.963	6.92E-07	1.18E-04	PVP01_1229400		lactate dehydrogenase, putative
exon_PVP01_1145000-E1	1128	337	29.876	5136.756	61.470	3.027	0.612	4.946	7.56E-07	1.24E-04	PVP01_1145000		conserved Plasmodium protein, unknown function
exon_PVP01_0905300-E1	1851	207	11.183	1922.791	38.632	3.409	0.692	4.923	8.51E-07	1.30E-04	PVP01_0905300		WD repeat-containing protein, putative
exon_PVP01_1216000-E1	1977	426	21.548	3704.854	74.796	2.737	0.556	4.926	8.41E-07	1.30E-04	PVP01_1216000		conserved protein, unknown function
exon_PVP01_0904300-E1	1758	251	14.278	2454.839	45.495	3.229	0.658	4.906	9.29E-07	1.37E-04	PVP01_0904300		CPW-WPC family protein
exon_PVP01_1235000-E1	651	301	46.237	7949.753	52.222	2.577	0.528	4.881	1.05E-06	1.51E-04	PVP01_1235000		conserved Plasmodium protein, unknown function
exon_PVP01_0931600-E1	1530	125	8.170	1404.710	22.800	3.347	0.691	4.845	1.27E-06	1.75E-04	PVP01_0931600		armadillo repeat protein PF16, putative
exon_PVP01_1137600-E1	2187	345	15.775	2712.305	59.931	2.722	0.567	4.798	1.60E-06	2.15E-04	PVP01_1137600	BTP1	basal complex transmembrane protein 1, putative
exon_PVP01_0702600-E1	915	1075	117.486	20200.192	190.828	2.756	0.576	4.780	1.75E-06	2.18E-04	PVP01_0702600	PH	PH domain-containing protein, putative
exon_PVP01_0716400-E1	1188	2067	173.990	29915.218	348.084	2.285	0.479	4.771	1.83E-06	2.18E-04	PVP01_0716400	GAP50	glideosome-associated protein 50, putative
exon_PVP01_1203200-E1	1506	469	31.142	5354.464	81.266	2.347	0.492	4.772	1.82E-06	2.18E-04	PVP01_1203200	GK	glycerol kinase, putative
exon_PVP01_1318300-E1	1053	785	74.549	12817.680	144.264	3.290	0.688	4.785	1.71E-06	2.18E-04	PVP01_1318300	PIP2	PhIL1 interacting protein PIP2, putative
exon_PVP01_1467200-E1	7743	1272	16.428	2824.529	228.328	3.199	0.671	4.767	1.87E-06	2.18E-04	PVP01_1467200	G377	osmiophilic body protein G377, putative
exon_PVP01_0607900-E1	1035	186	17.971	3089.874	32.047	2.849	0.601	4.743	2.11E-06	2.39E-04	PVP01_0607900		phospholipid scramblase, putative
exon_PVP01_1326800-E1	936	304	32.479	5584.263	52.072	2.478	0.524	4.727	2.28E-06	2.53E-04	PVP01_1326800		conserved Plasmodium protein, unknown function
exon_PVP01_0508500-E1	1296	221	17.052	2931.942	39.187	2.782	0.594	4.684	2.82E-06	3.04E-04	PVP01_0508500		conserved protein, unknown function
exon_PVP01_0836100-E1	1593	347	21.783	3745.259	60.218	2.567	0.551	4.660	3.16E-06	3.16E-04	PVP01_0836100	IMC1e	inner membrane complex protein 1e, putative
exon_PVP01_1020200-E1	2196	294	13.388	2301.882	52.658	2.842	0.609	4.670	3.02E-06	3.16E-04	PVP01_1020200	PSOP12	secreted ookinete protein, putative
exon_PVP01_1208000-E1	1302	1294	99.386	17088.008	229.586	3.062	0.657	4.657	3.21E-06	3.16E-04	PVP01_1208000	P47	6-cysteine protein
exon_PVP01_1301800-E1	3120	136	4.359	749.467	23.511	2.943	0.632	4.657	3.20E-06	3.16E-04	PVP01_1301800	TGS1	trimethylguanosine synthase, putative
exon_PVP01_1344600-E2	399	276	69.173	11893.353	51.223	3.177	0.687	4.625	3.75E-06	3.60E-04	PVP01_1344600		conserved Plasmodium protein, unknown function
exon_PVP01_1438100-E1	576	137	23.785	4089.462	23.996	2.860	0.619	4.621	3.81E-06	3.60E-04	PVP01_1438100	RKIP	raf kinase inhibitor, putative
exon_PVP01_0505700-E1	3135	468	14.928	2566.708	85.836	2.886	0.634	4.551	5.33E-06	4.65E-04	PVP01_0505700		conserved protein, unknown function
exon_PVP01_0816600-E3	2130	70	3.286	565.050	12.504	3.512	0.771	4.552	5.31E-06	4.65E-04	PVP01_0816600		conserved Plasmodium protein, unknown function
exon_PVP01_1115900-E1	4173	168	4.026	692.196	29.847	2.756	0.606	4.550	5.36E-06	4.65E-04	PVP01_1115900		conserved Plasmodium protein, unknown function
exon_PVP01_1425600-E1	1107	113	10.208	1755.088	19.658	3.138	0.688	4.562	5.06E-06	4.65E-04	PVP01_1425600		conserved Plasmodium protein, unknown function
exon_PVP01_1020200-E2	2196	983	44.763	7696.430	174.074	2.925	0.649	4.510	6.48E-06	5.41E-04	PVP01_1020200		secreted ookinete protein, putative
exon_PVP01_1270400-E1	4881	186	3.811	655.198	33.289	2.886	0.640	4.511	6.45E-06	5.41E-04	PVP01_1270400	CCp1	LCCL domain-containing protein
exon_PVP01_1128100-E1	1824	484	26.535	4562.351	87.066	2.789	0.619	4.502	6.73E-06	5.44E-04	PVP01_1128100	ALV7	inner membrane complex protein 1j, putative
exon_PVP01_1229400-E1	1005	358	35.622	6124.704	65.899	2.942	0.654	4.501	6.76E-06	5.44E-04	PVP01_1229400		lactate dehydrogenase, putative
exon_PVP01_0408100-E1	1521	113	7.429	1277.372	19.962	2.847	0.636	4.475	7.65E-06	5.62E-04	PVP01_0408100		conserved Plasmodium protein, unknown function
exon_PVP01_0712800-E6	750	169	22.533	3874.303	29.525	2.666	0.598	4.459	8.24E-06	5.62E-04	PVP01_0712800		conserved protein, unknown function
exon_PVP01_0932300-E1	777	703	90.476	15556.161	122.481	2.551	0.571	4.470	7.83E-06	5.62E-04	PVP01_0932300		conserved Plasmodium protein, unknown function
exon_PVP01_0937000-E1	1665	690	41.441	7125.297	120.660	2.537	0.565	4.489	7.17E-06	5.62E-04	PVP01_0937000		conserved Plasmodium protein, unknown function
exon_PVP01_0944200-E1	2958	243	8.215	1412.460	44.635	2.838	0.637	4.459	8.25E-06	5.62E-04	PVP01_0944200		conserved Plasmodium protein, unknown function
exon_PVP01_1017500-E6	1329	96	7.223	1241.979	17.011	3.066	0.685	4.477	7.56E-06	5.62E-04	PVP01_1017500	MFS1	major facilitator superfamily domain-containing protein, putative

Exon ID	TL	Reads count	CVGE	RPKM	Mean counts	Log2FC	SD	Wald stat	p-value	q-value	Gene ID	Symbol	Product description
exon_PVP01_1218300-E1	1137	173	15.215	2616.097	31.540	3.038	0.680	4.465	7.99E-06	5.62E-04	PVP01_1218300		conserved Plasmodium protein, unknown function
exon_PVP01_1225300-E1	954	58	6.080	1045.316	10.909	3.805	0.851	4.469	7.87E-06	5.62E-04	PVP01_1225300		conserved Plasmodium protein, unknown function
exon_PVP01_1323800-E1	561	235	41.889	7202.332	42.826	2.804	0.626	4.480	7.47E-06	5.62E-04	PVP01_1323800		conserved Plasmodium protein, unknown function
exon_PVP01_1421800-E1	1995	324	16.241	2792.353	58.072	2.808	0.629	4.462	8.11E-06	5.62E-04	PVP01_1421800		conserved Plasmodium protein, unknown function
exon_PVP01_0115200-E1	3600	83	2.306	396.409	14.881	3.351	0.754	4.445	8.81E-06	5.91E-04	PVP01_0115200		conserved Plasmodium protein, unknown function
exon_PVP01_0532000-E2	1116	186	16.667	2865.609	31.703	2.579	0.583	4.422	9.79E-06	6.29E-04	PVP01_0532000	GAPM2	glideosome associated protein with multiple membrane spans 2, putative
exon_PVP01_1230900-E3	912	176	19.298	3318.073	29.779	2.688	0.608	4.424	9.69E-06	6.29E-04	PVP01_1230900	GAPM1	glideosome associated protein with multiple membrane spans 1, putative
exon_PVP01_1253500-E1	3543	197	5.560	956.012	35.020	2.663	0.602	4.423	9.73E-06	6.29E-04	PVP01_1253500	NNT	NAD(P) transhydrogenase, putative
exon_PVP01_1208100-E1	1353	671	49.593	8526.933	121.812	2.825	0.640	4.416	1.01E-05	6.37E-04	PVP01_1208100	P48/45	6-cysteine protein
exon_PVP01_1018300-E1	4116	49	1.190	204.686	9.063	3.837	0.872	4.402	1.07E-05	6.68E-04	PVP01_1018300		conserved Plasmodium protein, unknown function
exon_PVP01_1022900-E2	366	212	57.923	9959.165	35.660	2.369	0.538	4.399	1.09E-05	6.68E-04	PVP01_1022900		conserved Plasmodium protein, unknown function
exon_PVP01_0605400-E3	2658	113	4.251	730.957	19.844	2.928	0.668	4.383	1.17E-05	6.92E-04	PVP01_0605400		conserved Plasmodium protein, unknown function
exon_PVP01_0942600-E1	1551	461	29.723	5110.428	82.219	2.661	0.607	4.385	1.16E-05	6.92E-04	PVP01_0942600	IMC1b	inner membrane complex protein 1b, putative
exon_PVP01_1457500-E1	576	651	113.021	19432.409	113.419	2.659	0.606	4.388	1.14E-05	6.92E-04	PVP01_1457500		conserved protein, unknown function
exon_PVP01_0530800-E1	1353	277	20.473	3520.060	49.989	2.856	0.653	4.375	1.22E-05	7.06E-04	PVP01_0530800		alpha tubulin 2, putative
exon_PVP01_1342100-E1	3681	139	3.776	649.258	18.812	-3.719	0.851	-4.373	1.23E-05	7.06E-04	PVP01_1342100	ClpC	ATP-dependent Clp protease regulatory subunit ClpC, putative
exon_PVP01_0313300-E1	1590	433	27.233	4682.296	77.677	2.719	0.624	4.359	1.31E-05	7.42E-04	PVP01_0313300	CDPK4	calcium-dependent protein kinase 4, putative
exon_PVP01_0508400-E1	1875	127	6.773	1164.583	22.568	2.631	0.605	4.346	1.39E-05	7.52E-04	PVP01_0508400		conserved Plasmodium protein, unknown function
exon_PVP01_1018500-E6	1815	260	14.325	2463.003	45.785	2.477	0.569	4.352	1.35E-05	7.52E-04	PVP01_1018500		conserved protein, unknown function
exon_PVP01_1258000-E3	750	239	31.867	5479.044	43.300	2.807	0.646	4.344	1.40E-05	7.52E-04	PVP01_1258000	GEST	gamete egress and sporozoite traversal protein, putative
exon_PVP01_1314300-E4	1149	202	17.581	3022.731	35.877	2.815	0.648	4.342	1.41E-05	7.52E-04	PVP01_1314300		conserved protein, unknown function
exon_PVP01_1328100-E1	774	330	42.636	7330.627	60.845	2.835	0.652	4.346	1.39E-05	7.52E-04	PVP01_1328100		dynein light chain, putative
exon_PVP01_1018200-E1	1395	624	44.731	7690.924	107.699	2.545	0.587	4.338	1.44E-05	7.59E-04	PVP01_1018200	GAP40	glideosome-associated protein 40, putative
exon_PVP01_0521000-E8	1086	176	16.206	2786.448	31.360	2.557	0.591	4.325	1.52E-05	7.95E-04	PVP01_0521000		RNA-binding protein, putative
exon_PVP01_0820000-E1	1683	341	20.261	3483.681	60.233	2.700	0.627	4.306	1.66E-05	8.57E-04	PVP01_0820000		CPW-WPC family protein
exon_PVP01_0945500-E1	1734	195	11.246	1933.542	34.109	2.774	0.645	4.298	1.72E-05	8.77E-04	PVP01_0945500		conserved Plasmodium protein, unknown function
exon_PVP01_1255400-E4	2625	160	6.095	1047.994	28.691	2.902	0.676	4.293	1.77E-05	8.89E-04	PVP01_1255400		LCCL domain-containing protein, putative
exon_PVP01_1259400-E1	360	160	44.444	7641.623	28.851	2.661	0.621	4.283	1.84E-05	9.16E-04	PVP01_1259400		conserved protein, unknown function
exon_PVP01_1032700-E1	957	105	10.972	1886.451	18.411	2.816	0.658	4.280	1.87E-05	9.19E-04	PVP01_1032700		conserved Plasmodium protein, unknown function
exon_PVP01_0507200-E1	3621	253	6.987	1201.324	44.986	2.517	0.591	4.257	2.07E-05	1.01E-03	PVP01_0507200		conserved Plasmodium protein, unknown function
exon_PVP01_1219900-E1	1236	514	41.586	7150.111	91.386	2.487	0.585	4.248	2.15E-05	1.03E-03	PVP01_1219900		MSP7-like protein
exon_PVP01_1432300-E1	1980	75	3.788	651.275	13.487	3.020	0.711	4.249	2.14E-05	1.03E-03	PVP01_1432300		integral membrane protein GPR180, putative
exon_PVP01_0311000-E1	498	403	80.924	13913.739	69.670	2.414	0.571	4.230	2.34E-05	1.08E-03	PVP01_0311000		conserved Plasmodium protein, unknown function
exon_PVP01_1249600-E1	1893	260	13.735	2361.516	45.326	2.266	0.535	4.233	2.31E-05	1.08E-03	PVP01_1249600		MA3 domain-containing protein, putative
exon_PVP01_1323800-E2	561	221	39.394	6773.257	39.862	2.717	0.643	4.229	2.35E-05	1.08E-03	PVP01_1323800		conserved Plasmodium protein, unknown function
exon_PVP01_0946800-E1	528	124	23.485	4037.903	21.631	2.791	0.662	4.217	2.47E-05	1.13E-03	PVP01_0946800		conserved protein, unknown function
exon_PVP01_0215500-E1	294	237	80.612	13860.189	42.937	2.516	0.597	4.214	2.51E-05	1.14E-03	PVP01_0215500		dynein light chain, putative
exon_PVP01_1433300-E1	564	215	38.121	6554.318	37.318	2.841	0.675	4.210	2.55E-05	1.14E-03	PVP01_1433300		conserved Plasmodium protein, unknown function
exon_PVP01_0814500-E1	978	127	12.986	2232.714	18.297	-3.354	0.798	-4.202	2.64E-05	1.16E-03	PVP01_0814500	MRK	MO15-related protein kinase, putative
exon_PVP01_1423200-E1	1905	200	10.499	1805.108	35.737	2.711	0.645	4.203	2.63E-05	1.16E-03	PVP01_1423200		MAATS1 domain-containing protein, putative
exon_PVP01_0919500-E1	750	127	16.933	2911.458	22.393	2.751	0.656	4.194	2.74E-05	1.19E-03	PVP01_0919500		conserved protein, unknown function
exon_PVP01_1407400-E1	12249	237	1.935	332.672	42.894	2.652	0.633	4.189	2.80E-05	1.21E-03	PVP01_1407400		MORN repeat protein, putative
exon_PVP01_1424500-E1	675	229	33.926	5833.106	39.856	2.381	0.570	4.181	2.90E-05	1.23E-03	PVP01_1424500	PSOP20	secreted ookinete protein, putative
exon_PVP01_0104000-E1	3123	362	11.591	1992.988	64.202	2.505	0.600	4.176	2.97E-05	1.25E-03	PVP01_0104000		conserved Plasmodium protein, unknown function
exon_PVP01_0904800-E2	1854	134	7.228	1242.691	24.316	2.888	0.692	4.172	3.01E-05	1.26E-03	PVP01_0904800		phenylalanine--tRNA ligase beta subunit, putative
exon_PVP01_1306800-E4	1122	165	14.706	2528.478	28.854	2.623	0.630	4.165	3.12E-05	1.29E-03	PVP01_1306800		41K blood stage antigen precursor 41-3, putative
exon_PVP01_0521000-E1	1086	112	10.313	1773.194	20.357	2.922	0.704	4.153	3.28E-05	1.33E-03	PVP01_0521000		RNA-binding protein, putative
exon_PVP01_1459500-E4	1362	135	9.912	1704.217	24.367	2.946	0.709	4.154	3.26E-05	1.33E-03	PVP01_1459500		GAS8-like protein, putative
exon_PVP01_1235200-E1	3600	96	2.667	458.497	16.854	2.722	0.656	4.150	3.32E-05	1.34E-03	PVP01_1235200		conserved Plasmodium protein, unknown function
exon_PVP01_1335700-E4	1293	237	18.329	3151.505	41.874	2.458	0.593	4.147	3.37E-05	1.35E-03	PVP01_1335700		conserved Plasmodium protein, unknown function
exon_PVP01_1258000-E1	750	51	6.800	1169.168	9.224	3.383	0.817	4.142	3.44E-05	1.36E-03	PVP01_1258000		gamete egress and sporozoite traversal protein, putative
exon_PVP01_0309800-E1	618	450	72.816	12519.650	77.858	2.412	0.585	4.127	3.68E-05	1.44E-03	PVP01_0309800		conserved Plasmodium protein, unknown function
exon_PVP01_0317400-E1	1431	341	23.829	4097.160	60.406	2.478	0.602	4.119	3.81E-05	1.48E-03	PVP01_0317400	PSOP1	secreted ookinete protein, putative
exon_PVP01_0405900-E1	1437	649	45.164	7765.261	114.323	2.403	0.585	4.110	3.96E-05	1.52E-03	PVP01_0405900		saccharopine dehydrogenase, putative
exon_PVP01_1114600-E1	369	127	34.417	5917.598	23.750	3.284	0.800	4.105	4.04E-05	1.54E-03	PVP01_1114600		dynein light chain, putative
exon_PVP01_0816200-E1	717	173	24.128	4148.538	31.166	2.667	0.651	4.094	4.25E-05	1.59E-03	PVP01_0816200		conserved Plasmodium protein, unknown function
exon_PVP01_1332500-E4	1962	56	2.854	490.746	9.976	3.282	0.802	4.094	4.24E-05	1.59E-03	PVP01_1332500		conserved protein, unknown function
exon_PVP01_0611300-E1	537	78	14.525	2497.402	13.527	2.793	0.684	4.081	4.49E-05	1.67E-03	PVP01_0611300		conserved Plasmodium protein, unknown function
exon_PVP01_0310300-E1	2895	568	19.620	3373.400	100.559	2.397	0.589	4.069	4.72E-05	1.73E-03	PVP01_0310300		conserved Plasmodium protein, unknown function
exon_PVP01_1425700-E1	1182	502	42.470	7302.211	89.214	2.496	0.613	4.071	4.69E-05	1.73E-03	PVP01_1425700		conserved protein, unknown function
exon_PVP01_0211500-E1	1335	185	13.858	2382.641	26.229	-2.768	0.681	-4.061	4.88E-05	1.77E-03	PVP01_0211500	NT4	nucleoside transporter 4
exon_PVP01_0944300-E1	975	355	36.410	6260.253	65.084	2.671	0.658	4.060	4.90E-05	1.77E-03	PVP01_0944300		conserved Plasmodium protein, unknown function
exon_PVP01_1025600-E4	1107	1047	94.580	16261.747	190.919	2.661	0.657	4.050	5.11E-05	1.81E-03	PVP01_1025600		conserved protein, unknown function
exon_PVP01_1439700-E1	1692	524	30.969	5324.748	92.498	2.587	0.638	4.051	5.09E-05	1.81E-03	PVP01_1439700	IMC1h	inner membrane complex protein 1h, putative
exon_PVP01_1447500-E2	351	598	170.370	29292.889	89.243	-2.359	0.583	-4.046	5.21E-05	1.83E-03	PVP01_1447500	MIF	macrophage migration inhibitory factor, putative

Exon ID	TL	Reads count	CVGE	RPKM	Mean counts	Log2FC	SD	Wald stat	p-value	q-value	Gene ID	Symbol	Product description
exon_PVP01_1104800-E1	1185	170	14.346	2466.600	30.918	2.565	0.636	4.035	5.45E-05	1.90E-03	PVP01_1104800		CLAMP domain-containing protein, putative
exon_PVP01_1342200-E1	4371	210	4.804	826.051	37.456	2.358	0.585	4.034	5.48E-05	1.90E-03	PVP01_1342200	WDR65	WD repeat-containing protein 65, putative
exon_PVP01_0511800-E3	597	64	10.720	1843.206	11.280	2.977	0.741	4.017	5.89E-05	1.94E-03	PVP01_0511800	DLC1	dynein light chain 1, putative
exon_PVP01_0530800-E2	1353	54	3.991	686.221	9.554	3.400	0.846	4.016	5.91E-05	1.94E-03	PVP01_0530800		alpha tubulin 2, putative
exon_PVP01_0929100-E1	4326	76	1.757	302.061	13.460	2.801	0.696	4.024	5.73E-05	1.94E-03	PVP01_0929100	PFK11	ATP-dependent 6-phosphofructokinase, putative
exon_PVP01_1017500-E5	1329	671	50.489	8680.918	119.378	2.577	0.642	4.015	5.94E-05	1.94E-03	PVP01_1017500		major facilitator superfamily domain-containing protein, putative
exon_PVP01_1113300-E1	2898	83	2.864	492.434	14.533	3.136	0.780	4.020	5.82E-05	1.94E-03	PVP01_1113300		zinc finger protein, putative
exon_PVP01_1245100-E4	402	76	18.905	3250.541	13.183	2.746	0.683	4.021	5.79E-05	1.94E-03	PVP01_1245100		conserved Plasmodium protein, unknown function
exon_PVP01_1266500-E1	1527	323	21.153	3636.902	58.108	2.306	0.573	4.023	5.76E-05	1.94E-03	PVP01_1266500		allantoicase, putative
exon_PVP01_1434600-E1	1215	672	55.309	9509.575	122.265	2.687	0.669	4.015	5.94E-05	1.94E-03	PVP01_1434600	RSPH9	radial spoke head protein 9, putative
exon_PVP01_0510800-E1	1887	375	19.873	3416.863	66.044	2.320	0.579	4.005	6.20E-05	2.01E-03	PVP01_0510800	IMC1i	inner membrane complex protein 1i, putative
exon_PVP01_0713600-E1	1068	595	55.712	9578.860	105.761	2.337	0.584	3.999	6.36E-05	2.04E-03	PVP01_0713600		ribonuclease H2 subunit C, putative
exon_PVP01_0517400-E1	300	876	292.000	50205.464	151.063	2.289	0.573	3.997	6.41E-05	2.04E-03	PVP01_0517400	HMGB2	high mobility group protein B2, putative
exon_PVP01_1124400-E1	1203	116	9.643	1657.908	20.165	2.524	0.632	3.995	6.47E-05	2.05E-03	PVP01_1124400	SMS2	sphingomyelin synthase 2, putative
exon_PVP01_1335900-E1	1323	298	22.525	3872.795	54.889	2.683	0.674	3.984	6.78E-05	2.13E-03	PVP01_1335900		conserved Plasmodium protein, unknown function
exon_PVP01_1312300-E1	2547	1778	69.808	12002.479	319.300	2.606	0.655	3.979	6.93E-05	2.16E-03	PVP01_1312300		ribonucleoside-diphosphate reductase large subunit, putative
exon_PVP01_0917100-E1	1356	396	29.204	5021.155	67.507	2.080	0.525	3.965	7.33E-05	2.27E-03	PVP01_0917100	FT2	folate transporter 2, putative
exon_PVP01_0206900-E2	657	412	62.709	10782.016	58.318	-2.376	0.600	-3.961	7.45E-05	2.28E-03	PVP01_0206900		proteasome subunit beta type-3, putative
exon_PVP01_1304700-E7	1005	281	27.960	4807.379	50.316	2.366	0.597	3.962	7.44E-05	2.28E-03	PVP01_1304700		tetratricopeptide repeat protein, putative
exon_PVP01_0618900-E8	1440	177	12.292	2113.386	31.729	2.689	0.679	3.959	7.54E-05	2.29E-03	PVP01_0618900		aspartyl protease, putative
exon_PVP01_1410400-E1	3318	408	12.297	2114.228	70.618	2.250	0.569	3.957	7.59E-05	2.29E-03	PVP01_1410400		HORMA domain protein, putative
exon_PVP01_0204200-E1	582	127	21.821	3751.879	18.940	-3.308	0.837	-3.951	7.80E-05	2.32E-03	PVP01_0204200	AKLP1	adenylate kinase-like protein 1, putative
exon_PVP01_0316800-E1	717	753	105.021	18056.932	103.522	-2.255	0.571	-3.951	7.77E-05	2.32E-03	PVP01_0316800		conserved protein, unknown function
exon_PVP01_0419500-E1	1998	279	13.964	2400.915	49.623	2.261	0.573	3.945	7.98E-05	2.36E-03	PVP01_0419500		conserved Plasmodium protein, unknown function
exon_PVP01_0723600-E1	1278	217	16.980	2919.423	38.529	2.603	0.660	3.941	8.10E-05	2.38E-03	PVP01_0723600		conserved Plasmodium protein, unknown function
exon_PVP01_1204000-E1	846	811	95.863	16482.331	141.527	2.354	0.598	3.935	8.32E-05	2.42E-03	PVP01_1204000		conserved Plasmodium protein, unknown function
exon_PVP01_1437500-E1	1593	62	3.892	669.182	11.003	2.999	0.764	3.924	8.69E-05	2.52E-03	PVP01_1437500		conserved Plasmodium protein, unknown function
exon_PVP01_0506400-E3	807	235	29.120	5006.826	41.241	2.427	0.620	3.916	8.99E-05	2.59E-03	PVP01_0506400	ROM3	rhomboid protease ROM3, putative
exon_PVP01_1115400-E2	594	83	13.973	2402.480	14.653	2.630	0.673	3.911	9.19E-05	2.63E-03	PVP01_1115400		conserved Plasmodium protein, unknown function
exon_PVP01_1020900-E1	816	720	88.235	15170.870	126.326	2.464	0.631	3.907	9.34E-05	2.65E-03	PVP01_1020900		conserved protein, unknown function
exon_PVP01_0108900-E2	759	413	54.414	9355.703	71.286	2.103	0.540	3.892	9.96E-05	2.81E-03	PVP01_0108900	IMC1d	inner membrane complex protein 1d, putative
exon_PVP01_1241300-E1	8149	36	0.442	75.957	6.711	3.671	0.944	3.890	1.00E-04	2.81E-03	PVP01_1241300		potassium channel K2, putative
exon_PVP01_1437600-E8	1053	104	9.877	1698.138	18.699	2.564	0.661	3.879	1.05E-04	2.92E-03	PVP01_1437600	PSOP17	secreted ookinete protein, putative
exon_PVP01_1119300-E5	402	42	10.448	1796.352	7.602	3.397	0.877	3.872	1.08E-04	2.99E-03	PVP01_1119300	PSOP6	secreted ookinete protein, putative
exon_PVP01_1454500-E2	1890	95	5.026	864.231	13.398	-3.305	0.857	-3.859	1.14E-04	3.13E-03	PVP01_1454500		conserved Plasmodium protein, unknown function
exon_PVP01_1225200-E1	1602	615	38.390	6600.559	103.890	2.077	0.539	3.854	1.16E-04	3.18E-03	PVP01_1225200	CAF2	chromatin assembly factor 1 subunit B, putative
exon_PVP01_1270600-E1	1521	183	12.032	2068.664	33.405	2.746	0.714	3.847	1.20E-04	3.25E-03	PVP01_1270600		tubulin epsilon chain, putative
exon_PVP01_0318500-E1	1659	570	34.358	5907.403	101.751	2.518	0.656	3.840	1.23E-04	3.28E-03	PVP01_0318500		conserved protein, unknown function
exon_PVP01_1123000-E1	2718	246	9.051	1556.158	44.040	2.314	0.602	3.841	1.23E-04	3.28E-03	PVP01_1123000		conserved Plasmodium protein, unknown function
exon_PVP01_1456100-E2	450	78	17.333	2980.233	13.773	2.643	0.688	3.841	1.23E-04	3.28E-03	PVP01_1456100		COPI associated protein, putative
exon_PVP01_0519000-E10	2649	144	5.436	934.649	26.428	2.682	0.701	3.826	1.30E-04	3.46E-03	PVP01_0519000		conserved Plasmodium protein, unknown function
exon_PVP01_0714200-E1	654	156	23.853	4101.238	27.296	2.382	0.623	3.823	1.32E-04	3.47E-03	PVP01_0714200	GLTP	glycolipid transfer protein, putative
exon_PVP01_0911900-E1	1050	940	89.524	15392.412	165.228	2.186	0.572	3.822	1.32E-04	3.47E-03	PVP01_0911900	RFC5	replication factor C subunit 5, putative
exon_PVP01_0608900-E1	2082	219	10.519	1808.554	38.973	2.454	0.643	3.819	1.34E-04	3.48E-03	PVP01_0608900		conserved Plasmodium protein, unknown function
exon_PVP01_0708000-E1	1470	281	19.116	3286.678	49.819	2.527	0.662	3.818	1.34E-04	3.48E-03	PVP01_0708000	SPM1	subpellicular microtubule protein 1, putative
exon_PVP01_1448300-E1	1098	771	70.219	12073.138	134.701	2.461	0.646	3.812	1.38E-04	3.55E-03	PVP01_1448300	TLAP1	thioredoxin-like associated protein 1, putative
exon_PVP01_0315400-E3	411	531	129.197	22213.697	73.637	-2.198	0.579	-3.795	1.48E-04	3.76E-03	PVP01_0315400	RPS10	40S ribosomal protein S10, putative
exon_PVP01_1265700-E5	7302	114	1.561	268.430	19.146	2.355	0.621	3.795	1.48E-04	3.76E-03	PVP01_1265700		serine/threonine protein kinase, putative
exon_PVP01_0307500-E1	1752	506	28.881	4965.747	92.461	2.454	0.649	3.784	1.54E-04	3.79E-03	PVP01_0307500		conserved Plasmodium protein, unknown function
exon_PVP01_0313300-E2	1590	431	27.107	4660.669	77.036	2.546	0.672	3.787	1.53E-04	3.79E-03	PVP01_0313300		calcium-dependent protein kinase 4, putative
exon_PVP01_0623100-E2	3663	1666	45.482	7819.990	239.689	-2.035	0.537	-3.791	1.50E-04	3.79E-03	PVP01_0623100		Plasmodium exported protein, unknown function
exon_PVP01_0834500-E3	420	211	50.238	8637.763	29.448	-2.513	0.664	-3.786	1.53E-04	3.79E-03	PVP01_0834500		ubiquitin-conjugating enzyme E2, putative
exon_PVP01_1104500-E1	1089	333	30.579	5257.563	57.107	1.963	0.518	3.788	1.52E-04	3.79E-03	PVP01_1104500		NADH-cytochrome b5 reductase, putative
exon_PVP01_1264900-E5	330	111	33.636	5783.319	20.325	2.640	0.698	3.785	1.54E-04	3.79E-03	PVP01_1264900	RPA3	replication factor A protein 3, putative
exon_PVP01_1438400-E1	1629	72	4.420	759.940	12.557	2.665	0.705	3.783	1.55E-04	3.79E-03	PVP01_1438400		zinc finger protein, putative
exon_PVP01_0108700-E1	2247	2892	128.705	22129.080	419.378	-1.730	0.458	-3.780	1.57E-04	3.82E-03	PVP01_0108700	HSP90	heat shock protein 90, putative
exon_PVP01_0944900-E1	816	177	21.691	3729.505	31.576	2.519	0.667	3.776	1.59E-04	3.84E-03	PVP01_0944900		conserved Plasmodium protein, unknown function
exon_PVP01_1323000-E1	1833	461	25.150	4324.208	83.373	2.358	0.624	3.777	1.59E-04	3.84E-03	PVP01_1323000		dynein intermediate chain, putative
exon_PVP01_0728500-E1	1863	656	35.212	6054.233	116.030	2.261	0.600	3.772	1.62E-04	3.89E-03	PVP01_0728500		procollagen lysine 5-dioxygenase, putative
exon_PVP01_1023300-E1	1092	62	5.678	976.196	11.145	2.769	0.736	3.763	1.68E-04	4.00E-03	PVP01_1023300		conserved Plasmodium protein, unknown function
exon_PVP01_1234300-E1	660	83	12.576	2162.232	14.812	2.742	0.729	3.761	1.69E-04	4.00E-03	PVP01_1234300		conserved Plasmodium protein, unknown function
exon_PVP01_1114900-E1	1332	528	39.640	6815.502	75.611	-1.990	0.529	-3.760	1.70E-04	4.01E-03	PVP01_1114900		elongation factor 1-alpha, putative
exon_PVP01_0608300-E1	15804	252	1.595	274.158	45.675	2.359	0.630	3.746	1.80E-04	4.19E-03	PVP01_0608300		dynein heavy chain, putative
exon_PVP01_1244800-E1	366	116	31.694	5449.354	20.714	2.552	0.681	3.747	1.79E-04	4.19E-03	PVP01_1244800	TLP1	thioredoxin-like protein
exon_PVP01_1026500-E1	1893	243	12.837	2207.109	42.895	2.241	0.599	3.740	1.84E-04	4.26E-03	PVP01_1026500	SUB1	subtilisin-like protease 1

Exon ID	TL	Reads count	CVGE	RPKM	Mean counts	Log2FC	SD	Wald stat	p-value	q-value	Gene ID	Symbol	Product description
exon_PVP01_1139400-E10	3258	108	3.315	569.955	20.038	2.730	0.730	3.738	1.86E-04	4.29E-03	PVP01_1139400		conserved Plasmodium protein, unknown function
exon_PVP01_1236900-E3	1290	228	17.674	3038.878	41.931	2.448	0.655	3.735	1.87E-04	4.30E-03	PVP01_1236900		protein MGET, putative
exon_PVP01_1416000-E1	1107	74	6.685	1149.350	13.842	3.247	0.870	3.733	1.89E-04	4.32E-03	PVP01_1416000	PK9	serine/threonine protein kinase PK9, putative
exon_PVP01_1306800-E9	1122	160	14.260	2451.858	28.226	2.350	0.630	3.730	1.91E-04	4.35E-03	PVP01_1306800		41K blood stage antigen precursor 41-3, putative
exon_PVP01_0115400-E1	1851	80	4.322	743.108	14.237	2.719	0.731	3.721	1.98E-04	4.47E-03	PVP01_0115400		conserved protein, unknown function
exon_PVP01_1309400-E1	3018	215	7.124	1224.863	38.207	2.198	0.591	3.721	1.99E-04	4.47E-03	PVP01_1309400		zinc finger protein, putative
exon_PVP01_1219100-E1	1500	106	7.067	1215.018	19.375	2.754	0.740	3.719	2.00E-04	4.47E-03	PVP01_1219100		DNA replication complex GINS protein, putative
exon_PVP01_1143500-E1	477	250	52.411	9011.348	44.977	2.435	0.655	3.716	2.03E-04	4.51E-03	PVP01_1143500		calcium-binding protein, putative
exon_PVP01_0825200-E1	1884	228	12.102	2080.760	39.297	2.183	0.590	3.699	2.16E-04	4.79E-03	PVP01_0825200		conserved Plasmodium protein, unknown function
exon_PVP01_1223200-E1	1446	638	44.122	7586.134	111.330	2.404	0.651	3.695	2.20E-04	4.85E-03	PVP01_1223200		CPW-WPC family protein
exon_PVP01_1419800-E1	1938	45	2.322	399.233	7.951	3.025	0.820	3.689	2.25E-04	4.94E-03	PVP01_1419800		conserved Plasmodium protein, unknown function
exon_PVP01_0527000-E3	2844	61	2.145	368.781	11.358	3.030	0.822	3.685	2.29E-04	4.99E-03	PVP01_0527000		conserved Plasmodium protein, unknown function
exon_PVP01_0941100-E1	498	109	21.888	3763.269	18.430	2.417	0.656	3.684	2.30E-04	4.99E-03	PVP01_0941100		conserved Plasmodium protein, unknown function
exon_PVP01_0810500-E2	1146	70	6.108	1050.223	12.757	2.744	0.746	3.680	2.34E-04	5.05E-03	PVP01_0810500		MORN repeat protein, putative
exon_PVP01_1240300-E6	390	98	25.128	4320.456	17.146	2.511	0.683	3.676	2.37E-04	5.10E-03	PVP01_1240300		conserved Plasmodium protein, unknown function
exon_PVP01_1214000-E2	318	78	24.528	4217.311	13.415	2.532	0.691	3.665	2.48E-04	5.28E-03	PVP01_1214000	MPC1	mitochondrial pyruvate carrier protein 1, putative
exon_PVP01_1300900-E1	891	1133	127.160	21863.533	205.786	2.499	0.682	3.665	2.47E-04	5.28E-03	PVP01_1300900	NEK3	NIMA related kinase 3, putative
exon_PVP01_0204400-E1	1344	1345	100.074	17206.445	234.161	1.827	0.499	3.662	2.50E-04	5.31E-03	PVP01_0204400		chromatin assembly factor 1 protein WD40 domain, putative
exon_PVP01_1228200-E1	810	176	21.728	3735.905	31.614	2.567	0.701	3.661	2.52E-04	5.31E-03	PVP01_1228200		conserved Plasmodium protein, unknown function
exon_PVP01_0938700-E1	2073	217	10.468	1799.818	39.540	2.370	0.648	3.659	2.54E-04	5.32E-03	PVP01_0938700		calcium-binding protein, putative
exon_PVP01_0814900-E7	2436	26	1.067	183.512	4.790	3.579	0.979	3.654	2.58E-04	5.39E-03	PVP01_0814900		conserved Plasmodium protein, unknown function
exon_PVP01_1205700-E1	612	184	30.065	5169.333	26.986	-2.392	0.657	-3.641	2.72E-04	5.66E-03	PVP01_1205700		nucleoside diphosphate hydrolase, putative
exon_PVP01_1311700-E1	558	149	26.703	4591.136	21.395	-2.773	0.764	-3.631	2.82E-04	5.84E-03	PVP01_1311700	TRAPPC5	trafficking protein particle complex subunit 5, putative
exon_PVP01_0211500-E2	1335	85	6.367	1094.727	12.131	-3.166	0.874	-3.624	2.90E-04	5.93E-03	PVP01_0211500		nucleoside transporter 4
exon_PVP01_1027600-E1	381	123	32.283	5550.707	21.689	2.436	0.672	3.624	2.91E-04	5.93E-03	PVP01_1027600		conserved Plasmodium protein, unknown function
exon_PVP01_1032200-E1	534	161	30.150	5183.854	22.489	-2.543	0.702	-3.625	2.89E-04	5.93E-03	PVP01_1032200		DnaJ protein, putative
exon_PVP01_1259400-E4	360	81	22.500	3868.572	14.061	2.452	0.677	3.621	2.93E-04	5.95E-03	PVP01_1259400		conserved protein, unknown function
exon_PVP01_0318800-E1	1218	63	5.172	889.327	11.292	2.784	0.769	3.619	2.96E-04	5.96E-03	PVP01_0318800	CPH1	protein CPH1, putative
exon_PVP01_0904800-E3	1854	70	3.776	649.167	12.992	2.954	0.816	3.619	2.96E-04	5.96E-03	PVP01_0904800		phenylalanine--tRNA ligase beta subunit, putative
exon_PVP01_0907700-E1	507	93	18.343	3153.865	16.260	2.852	0.789	3.616	3.00E-04	6.01E-03	PVP01_0907700		conserved Plasmodium protein, unknown function
exon_PVP01_1144500-E1	597	226	37.856	6508.820	39.454	2.138	0.592	3.611	3.04E-04	6.08E-03	PVP01_1144500		conserved protein, unknown function
exon_PVP01_0806000-E6	396	61	15.404	2648.517	10.929	3.009	0.834	3.607	3.09E-04	6.12E-03	PVP01_0806000		conserved Plasmodium protein, unknown function
exon_PVP01_1024300-E7	1191	92	7.725	1328.141	16.115	2.378	0.659	3.608	3.08E-04	6.12E-03	PVP01_1024300		conserved Plasmodium protein, unknown function
exon_PVP01_1341700-E1	3921	583	14.869	2556.465	103.953	2.259	0.626	3.606	3.11E-04	6.13E-03	PVP01_1341700	CCp3	LCCL domain-containing protein, putative
exon_PVP01_1228300-E1	1644	228	13.869	2384.521	40.005	2.500	0.694	3.604	3.13E-04	6.14E-03	PVP01_1228300		TBC domain containing protein
exon_PVP01_1227100-E1	5397	276	5.114	879.275	48.513	2.206	0.613	3.598	3.21E-04	6.26E-03	PVP01_1227100		conserved Plasmodium protein, unknown function
exon_PVP01_0309200-E7	1245	105	8.434	1450.067	18.652	2.312	0.644	3.590	3.30E-04	6.39E-03	PVP01_0309200	ALP5b	actin-like protein, putative
exon_PVP01_0922400-E1	5070	82	1.617	278.083	14.300	2.428	0.677	3.589	3.32E-04	6.39E-03	PVP01_0922400		peptidase M16, putative
exon_PVP01_1303300-E1	2556	112	4.382	753.399	19.813	2.358	0.657	3.590	3.31E-04	6.39E-03	PVP01_1303300		conserved Plasmodium protein, unknown function
exon_PVP01_1145300-E1	6984	189	2.706	465.292	32.841	2.001	0.559	3.579	3.45E-04	6.59E-03	PVP01_1145300		conserved Plasmodium protein, unknown function
exon_PVP01_1229800-E1	1401	257	18.344	3154.010	39.352	-2.110	0.589	-3.580	3.44E-04	6.59E-03	PVP01_1229800	DHFS-FPGS	dihydrofolate synthase/folylpolyglutamate synthase, putative
exon_PVP01_1016500-E1	2403	51	2.122	364.909	9.011	2.767	0.773	3.577	3.48E-04	6.61E-03	PVP01_1016500		UTP--glucose-1-phosphate uridylyltransferase, putative
exon_PVP01_0605500-E1	2208	166	7.518	1292.639	27.566	2.158	0.604	3.572	3.54E-04	6.70E-03	PVP01_0605500		cytoplasmic dynein intermediate chain, putative
exon_PVP01_1328100-E4	774	67	8.656	1488.339	12.251	2.944	0.824	3.571	3.56E-04	6.71E-03	PVP01_1328100		dynein light chain, putative
exon_PVP01_0821700-E1	1929	111	5.754	989.370	20.737	2.701	0.758	3.564	3.66E-04	6.87E-03	PVP01_0821700		conserved Plasmodium protein, unknown function
exon_PVP01_1121900-E1	1251	169	13.509	2322.724	29.577	2.144	0.602	3.562	3.69E-04	6.89E-03	PVP01_1121900		conserved Plasmodium protein, unknown function
exon_PVP01_1110100-E1	825	1425	172.727	29698.126	245.899	1.854	0.522	3.554	3.80E-04	7.04E-03	PVP01_1110100	PCNA1	proliferating cell nuclear antigen 1, putative
exon_PVP01_1230900-E1	912	106	11.623	1998.385	17.534	2.397	0.674	3.554	3.80E-04	7.04E-03	PVP01_1230900		glideosome associated protein with multiple membrane spans 1, putative
exon_PVP01_1224800-E1	1404	81	5.769	991.941	14.063	2.363	0.665	3.550	3.85E-04	7.08E-03	PVP01_1224800	ACBP1	apicoplast calcium binding protein 1, putative
exon_PVP01_1345800-E4	1347	109	8.092	1391.320	19.788	2.650	0.747	3.549	3.87E-04	7.08E-03	PVP01_1345800		conserved Plasmodium protein, unknown function
exon_PVP01_1408300-E1	1113	151	13.567	2332.652	27.130	2.457	0.692	3.550	3.85E-04	7.08E-03	PVP01_1408300		conserved Plasmodium protein, unknown function
exon_PVP01_1119300-E1	402	63	15.672	2694.528	11.037	2.605	0.735	3.546	3.91E-04	7.13E-03	PVP01_1119300		secreted ookinete protein, putative
exon_PVP01_1240300-E1	4350	78	1.793	308.300	13.808	2.727	0.770	3.543	3.96E-04	7.18E-03	PVP01_1240300	K2	potassium channel K2, putative
exon_PVP01_1028300-E1	1665	134	8.048	1383.753	23.673	2.303	0.651	3.536	4.06E-04	7.34E-03	PVP01_1028300		conserved Plasmodium protein, unknown function
exon_PVP01_0419900-E4	576	98	17.014	2925.309	18.011	2.556	0.724	3.531	4.14E-04	7.40E-03	PVP01_0419900		MORN repeat protein, putative
exon_PVP01_0726650-E1	342	267	78.070	13423.114	47.787	2.314	0.655	3.533	4.11E-04	7.40E-03	PVP01_0726650		conserved Plasmodium protein, unknown function
exon_PVP01_0907700-E2	507	63	12.426	2136.489	11.015	2.781	0.788	3.531	4.14E-04	7.40E-03	PVP01_0907700		conserved Plasmodium protein, unknown function
exon_PVP01_1262400-E2	375	71	18.933	3255.331	12.826	2.590	0.734	3.526	4.22E-04	7.51E-03	PVP01_1262400		conserved Plasmodium protein, unknown function
exon_PVP01_0809100-E1	1401	235	16.774	2884.017	41.610	2.374	0.674	3.524	4.25E-04	7.53E-03	PVP01_0809100		conserved Plasmodium protein, unknown function
exon_PVP01_1310200-E3	5046	76	1.506	258.961	13.167	2.373	0.674	3.523	4.26E-04	7.53E-03	PVP01_1310200		kinesin-7, putative
exon_PVP01_1214400-E3	1134	376	33.157	5700.893	65.484	2.244	0.638	3.516	4.38E-04	7.70E-03	PVP01_1214400		conserved Plasmodium protein, unknown function
exon_PVP01_1328100-E3	774	49	6.331	1088.487	8.842	2.792	0.794	3.515	4.40E-04	7.70E-03	PVP01_1328100		dynein light chain, putative
exon_PVP01_1262200-E2	1110	2390	215.315	37020.566	424.659	2.040	0.581	3.513	4.42E-04	7.72E-03	PVP01_1262200		fructose 1,6-bisphosphate aldolase, putative
exon_PVP01_0933100-E1	825	449	54.424	9357.515	78.166	1.980	0.565	3.506	4.55E-04	7.90E-03	PVP01_0933100		nucleic acid binding protein, putative
exon_PVP01_0808400-E2	1338	112	8.371	1439.229	19.392	2.292	0.654	3.504	4.58E-04	7.93E-03	PVP01_0808400		tubulin beta chain, putative

Exon ID	TL	Reads count	CVGE	RPKM	Mean counts	Log2FC	SD	Wald stat	p-value	q-value	Gene ID	Symbol	Product description
exon_PVP01_0828500-E1	1317	95	7.213	1240.241	16.340	2.283	0.652	3.503	4.60E-04	7.94E-03	PVP01_0828500	PM6	plasmepsin VI, putative
exon_PVP01_0518400-E1	780	519	66.538	11440.392	90.707	1.884	0.539	3.496	4.72E-04	8.10E-03	PVP01_0518400	AK2	adenylate kinase 2, putative
exon_PVP01_1024300-E2	1191	54	4.534	779.561	9.771	2.754	0.789	3.493	4.78E-04	8.18E-03	PVP01_1024300		conserved Plasmodium protein, unknown function
exon_PVP01_0209700-E1	441	145	32.880	5653.242	20.940	-2.436	0.699	-3.486	4.91E-04	8.30E-03	PVP01_0209700		conserved Plasmodium protein, unknown function
exon_PVP01_0307800-E1	1902	433	22.766	3914.223	73.959	2.043	0.586	3.486	4.90E-04	8.30E-03	PVP01_0307800	FEN1	flap endonuclease 1, putative
exon_PVP01_1335700-E1	1293	78	6.032	1037.204	14.508	2.884	0.827	3.486	4.91E-04	8.30E-03	PVP01_1335700		conserved Plasmodium protein, unknown function
exon_PVP01_0114400-E1	1512	117	7.738	1330.461	16.197	-2.651	0.762	-3.480	5.01E-04	8.44E-03	PVP01_0114400		conserved Plasmodium protein, unknown function
exon_PVP01_0834400-E1	2673	470	17.583	3023.201	82.954	1.975	0.568	3.479	5.04E-04	8.46E-03	PVP01_0834400		P-loop containing nucleoside triphosphate hydrolase, putative
exon_PVP01_0532000-E1	1116	377	33.781	5808.250	61.751	2.196	0.632	3.476	5.08E-04	8.50E-03	PVP01_0532000		glideosome associated protein with multiple membrane spans 2, putative
exon_PVP01_1436400-E1	1005	174	17.313	2976.811	31.839	2.573	0.741	3.474	5.13E-04	8.54E-03	PVP01_1436400	APC10	anaphase-promoting complex subunit 10, putative
exon_PVP01_1408300-E2	1113	207	18.598	3197.741	37.575	2.554	0.738	3.463	5.34E-04	8.87E-03	PVP01_1408300		conserved Plasmodium protein, unknown function
exon_PVP01_0805800-E1	558	35	6.272	1078.455	6.068	3.033	0.877	3.460	5.40E-04	8.93E-03	PVP01_0805800	NOT5	CCR4-NOT transcription complex subunit 5, putative
exon_PVP01_0729500-E1	1416	44	3.107	534.266	8.021	2.988	0.864	3.457	5.47E-04	9.01E-03	PVP01_0729500		conserved Plasmodium protein, unknown function
exon_PVP01_0718700-E1	561	130	23.173	3984.269	22.274	2.435	0.705	3.454	5.51E-04	9.05E-03	PVP01_0718700		conserved Plasmodium protein, unknown function
exon_PVP01_0412200-E1	1104	97	8.786	1510.674	13.089	-2.884	0.836	-3.450	5.61E-04	9.14E-03	PVP01_0412200		SRR1-like protein
exon_PVP01_0702100-E3	1362	672	49.339	8483.212	108.618	1.584	0.459	3.449	5.63E-04	9.14E-03	PVP01_0702100		alpha tubulin 1, putative
exon_PVP01_0832900-E1	1728	116	6.713	1154.203	20.755	2.346	0.680	3.449	5.63E-04	9.14E-03	PVP01_0832900	EB1	microtubule-associated protein RP/EB family, putative
exon_PVP01_1117000-E1	2016	61	3.026	520.244	10.558	2.593	0.753	3.444	5.73E-04	9.26E-03	PVP01_1117000	MCA1	metacaspase-1, putative
exon_PVP01_0711000-E1	3477	136	3.911	672.516	25.295	2.562	0.744	3.441	5.79E-04	9.32E-03	PVP01_0711000		conserved Plasmodium protein, unknown function
exon_PVP01_1327200-E1	1176	175	14.881	2558.579	30.327	2.087	0.607	3.438	5.86E-04	9.37E-03	PVP01_1327200		conserved Plasmodium protein, unknown function
exon_PVP01_1432300-E2	1980	68	3.434	590.489	12.490	2.685	0.781	3.439	5.84E-04	9.37E-03	PVP01_1432300		integral membrane protein GPR180, putative
exon_PVP01_0112500-E2	1158	182	15.717	2702.284	25.978	-2.227	0.649	-3.429	6.06E-04	9.62E-03	PVP01_0112500	TyrRS	tyrosine--tRNA ligase, putative
exon_PVP01_0947100-E1	1023	194	18.964	3260.575	35.111	2.340	0.682	3.430	6.04E-04	9.62E-03	PVP01_0947100	LRR10	leucine-rich repeat protein
exon_PVP01_1322800-E1	2088	333	15.948	2742.091	58.100	2.048	0.598	3.422	6.21E-04	9.82E-03	PVP01_1322800	ABCG2	ABC transporter G family member 2, putative
exon_PVP01_0945100-E1	4296	155	3.608	620.348	28.260	2.224	0.651	3.419	6.29E-04	9.92E-03	PVP01_0945100		conserved Plasmodium protein, unknown function
exon_PVP01_0707600-E1	1884	142	7.537	1295.912	23.842	1.967	0.576	3.417	6.33E-04	9.93E-03	PVP01_0707600		conserved Plasmodium protein, unknown function
exon_PVP01_1216300-E1	1365	100	7.326	1259.608	18.626	2.612	0.765	3.416	6.34E-04	9.93E-03	PVP01_1216300		SprT-like domain-containing protein, putative
exon_PVP01_1112400-E1	567	86	15.168	2607.856	14.065	2.430	0.712	3.413	6.42E-04	9.98E-03	PVP01_1112400		conserved Plasmodium protein, unknown function
exon_PVP01_1456500-E1	903	96	10.631	1827.897	16.959	2.284	0.669	3.413	6.42E-04	9.98E-03	PVP01_1456500		conserved protein, unknown function
exon_PVP01_0726600-E2	1191	231	19.395	3334.789	40.931	2.151	0.631	3.409	6.51E-04	1.01E-02	PVP01_0726600		conserved Plasmodium protein, unknown function
exon_PVP01_0831500-E3	1350	126	9.333	1604.741	21.006	2.029	0.596	3.405	6.61E-04	1.02E-02	PVP01_0831500		conserved protein, unknown function
exon_PVP01_0905400-E1	1899	69	3.633	624.730	12.661	2.624	0.772	3.399	6.78E-04	1.04E-02	PVP01_0905400		radial spoke head protein, putative
exon_PVP01_0947300-E9	2403	79	3.288	565.251	14.480	2.530	0.744	3.399	6.76E-04	1.04E-02	PVP01_0947300		kinesin-like protein, putative
exon_PVP01_0603300-E1	810	250	30.864	5306.683	44.694	2.162	0.637	3.395	6.86E-04	1.05E-02	PVP01_0603300		conserved Plasmodium protein, unknown function
exon_PVP01_0826600-E1	1917	36	1.878	322.885	6.482	2.983	0.880	3.392	6.95E-04	1.05E-02	PVP01_0826600		conserved Plasmodium protein, unknown function
exon_PVP01_0833800-E1	1968	433	22.002	3782.953	73.720	1.824	0.538	3.391	6.95E-04	1.05E-02	PVP01_0833800		FAD-dependent glycerol-3-phosphate dehydrogenase, putative
exon_PVP01_1243500-E1	1644	353	21.472	3691.824	61.399	1.905	0.562	3.389	7.00E-04	1.06E-02	PVP01_1243500		DNA polymerase alpha subunit B, putative
exon_PVP01_0710100-E1	1485	157	10.572	1817.780	20.327	-2.530	0.747	-3.386	7.09E-04	1.07E-02	PVP01_0710100		conserved Plasmodium protein, unknown function
exon_PVP01_1310900-E1	2277	565	24.813	4266.321	96.602	1.867	0.553	3.379	7.29E-04	1.09E-02	PVP01_1310900	MCM5	DNA replication licensing factor MCM5, putative
exon_PVP01_0718700-E4	561	30	5.348	919.447	5.584	3.264	0.967	3.374	7.41E-04	1.11E-02	PVP01_0718700		conserved Plasmodium protein, unknown function
exon_PVP01_1408200-E1	1881	112	5.954	1023.758	17.001	-2.481	0.735	-3.374	7.40E-04	1.11E-02	PVP01_1408200	DBP6	ATP-dependent RNA helicase DBP6, putative
exon_PVP01_1336700-E2	2397	98	4.088	702.953	17.730	2.373	0.704	3.370	7.51E-04	1.12E-02	PVP01_1336700		conserved Plasmodium protein, unknown function
exon_PVP01_0407000-E1	993	623	62.739	10787.155	105.023	1.776	0.527	3.368	7.58E-04	1.12E-02	PVP01_0407000	RFC2	replication factor C subunit 2, putative
exon_PVP01_1424200-E2	297	38	12.795	2199.861	6.923	2.874	0.854	3.365	7.66E-04	1.13E-02	PVP01_1424200	MYCBP	C-Myc-binding protein, putative
exon_PVP01_0118000-E1	663	424	63.952	10995.639	61.199	-1.999	0.595	-3.361	7.76E-04	1.14E-02	PVP01_0118000	1-CysPrx	1-cys peroxiredoxin
exon_PVP01_0609600-E1	1248	131	10.497	1804.782	22.702	2.015	0.600	3.360	7.79E-04	1.14E-02	PVP01_0609600		conserved Plasmodium protein, unknown function
exon_PVP01_1216400-E2	1236	1036	83.819	14411.508	141.636	-1.767	0.526	-3.361	7.77E-04	1.14E-02	PVP01_1216400	EF-1gamma	elongation factor 1-gamma, putative
exon_PVP01_1433600-E1	330	148	44.848	7711.092	26.131	2.647	0.788	3.360	7.79E-04	1.14E-02	PVP01_1433600		conserved Plasmodium protein, unknown function
exon_PVP01_0730300-E2	765	46	6.013	1033.867	8.276	2.797	0.833	3.358	7.86E-04	1.14E-02	PVP01_0730300		conserved protein, unknown function
exon_PVP01_1424300-E1	4605	188	4.083	701.934	32.046	2.018	0.601	3.356	7.90E-04	1.14E-02	PVP01_1424300		conserved Plasmodium protein, unknown function
exon_PVP01_0810500-E1	1146	35	3.054	525.112	6.161	3.038	0.905	3.355	7.93E-04	1.14E-02	PVP01_0810500		MORN repeat protein, putative
exon_PVP01_0315200-E1	1302	455	34.946	6008.534	73.261	1.734	0.517	3.353	7.98E-04	1.15E-02	PVP01_0315200	CDC50A	LEM3/CDC50 family protein, putative
exon_PVP01_0319000-E1	1341	43	3.207	551.325	7.557	2.724	0.813	3.351	8.06E-04	1.15E-02	PVP01_0319000		conserved protein, unknown function
exon_PVP01_0935700-E1	1998	76	3.804	654.013	14.110	2.514	0.750	3.351	8.06E-04	1.15E-02	PVP01_0935700		conserved Plasmodium protein, unknown function
exon_PVP01_0836300-E3	588	78	13.265	2280.791	13.592	2.413	0.721	3.349	8.12E-04	1.16E-02	PVP01_0836300		phosphatidylethanolamine-binding protein, putative
exon_PVP01_1015100-E1	624	69	11.058	1901.221	12.407	2.398	0.717	3.346	8.19E-04	1.16E-02	PVP01_1015100	PSOP13	secreted ookinete protein, putative
exon_PVP01_0607600-E4	1635	268	16.391	2818.287	43.867	1.752	0.525	3.340	8.39E-04	1.18E-02	PVP01_0607600	SRSF4	serine/arginine-rich splicing factor 4, putative
exon_PVP01_1432200-E1	1761	78	4.429	761.559	13.821	2.414	0.723	3.340	8.39E-04	1.18E-02	PVP01_1432200		kelch domain-containing protein, putative
exon_PVP01_1029700-E1	678	34	5.015	862.219	6.113	3.040	0.911	3.338	8.45E-04	1.19E-02	PVP01_1029700		conserved Plasmodium protein, unknown function
exon_PVP01_1110700-E1	750	47	6.267	1077.469	8.259	2.617	0.786	3.328	8.76E-04	1.23E-02	PVP01_1110700		conserved Plasmodium protein, unknown function
exon_PVP01_1341900-E3	855	111	12.982	2232.158	18.610	2.098	0.631	3.327	8.79E-04	1.23E-02	PVP01_1341900	GAPM3	glideosome associated protein with multiple membrane spans 3, putative
exon_PVP01_0921200-E2	9624	149	1.548	266.194	26.456	2.453	0.738	3.325	8.86E-04	1.23E-02	PVP01_0921200		DDRGK domain-containing protein, putative
exon_PVP01_1459500-E1	1362	115	8.443	1451.740	20.752	2.352	0.708	3.323	8.89E-04	1.23E-02	PVP01_1459500		GAS8-like protein, putative
exon_PVP01_1250500-E2	2784	95	3.412	586.709	16.921	2.317	0.699	3.317	9.09E-04	1.26E-02	PVP01_1250500		conserved Plasmodium protein, unknown function
exon_PVP01_1231600-E2	726	83	11.433	1965.665	14.905	2.485	0.749	3.315	9.15E-04	1.26E-02	PVP01_1231600	FabZ	beta-hydroxyacyl-ACP dehydratase, putative

Exon ID	TL	Reads count	CVGE	RPKM	Mean counts	Log2FC	SD	Wald stat	p-value	q-value	Gene ID	Symbol	Product description
exon_PVP01_0604400-E1	510	65	12.745	2191.348	11.386	2.436	0.736	3.312	9.27E-04	1.28E-02	PVP01_0604400		conserved Plasmodium protein, unknown function
exon_PVP01_1234300-E3	660	36	5.455	937.836	6.415	2.832	0.856	3.308	9.41E-04	1.29E-02	PVP01_1234300		conserved Plasmodium protein, unknown function
exon_PVP01_1118700-E1	1164	59	5.069	871.500	10.872	2.913	0.881	3.306	9.47E-04	1.29E-02	PVP01_1118700		TPH domain-containing protein, putative
exon_PVP01_1102700-E1	1299	102	7.852	1350.079	18.046	2.451	0.742	3.304	9.52E-04	1.30E-02	PVP01_1102700		conserved Plasmodium protein, unknown function
exon_PVP01_1418900-E1	570	103	18.070	3106.923	17.393	2.181	0.660	3.304	9.53E-04	1.30E-02	PVP01_1418900		conserved protein, unknown function
exon_PVP01_0702800-E1	558	139	24.910	4283.007	20.287	-2.240	0.679	-3.301	9.65E-04	1.31E-02	PVP01_0702800	SPC3	signal peptidase complex subunit 3, putative
exon_PVP01_1340600-E1	3312	238	7.186	1235.534	42.531	2.042	0.619	3.298	9.75E-04	1.32E-02	PVP01_1340600	FANCJ	FANCJ-like helicase, putative
exon_PVP01_0928400-E1	1182	49	4.146	712.766	9.154	2.784	0.845	3.296	9.81E-04	1.32E-02	PVP01_0928400		conserved Plasmodium protein, unknown function
exon_PVP01_1214300-E1	3561	105	2.949	506.974	18.248	2.036	0.618	3.294	9.88E-04	1.33E-02	PVP01_1214300		conserved Plasmodium protein, unknown function
exon_PVP01_0823600-E1	1365	630	46.154	7935.532	105.772	1.603	0.487	3.289	1.00E-03	1.34E-02	PVP01_0823600	NUF2	kinetochore protein NUF2, putative
exon_PVP01_1325400-E1	855	61	7.135	1226.682	10.897	2.403	0.731	3.290	1.00E-03	1.34E-02	PVP01_1325400		calcium-dependent protein kinase, putative
exon_PVP01_0414700-E1	6750	256	3.793	652.085	44.518	2.275	0.692	3.286	1.02E-03	1.35E-02	PVP01_0414700		conserved Plasmodium protein, unknown function
exon_PVP01_0117200-E1	1773	356	20.079	3452.307	58.918	1.647	0.502	3.282	1.03E-03	1.37E-02	PVP01_0117200	CYP81	peptidyl-prolyl cis-trans isomerase, putative
exon_PVP01_0114300-E10	3153	45	1.427	245.390	7.988	2.751	0.838	3.281	1.03E-03	1.37E-02	PVP01_0114300	DPY19	Dpy-19-like C-mannosyltransferase, putative
exon_PVP01_1328400-E1	3066	180	5.871	1009.412	30.711	1.806	0.551	3.277	1.05E-03	1.38E-02	PVP01_1328400		protein GPR89, putative
exon_PVP01_1249800-E1	2433	167	6.864	1180.164	30.147	2.460	0.751	3.274	1.06E-03	1.40E-02	PVP01_1249800		conserved Plasmodium protein, unknown function
exon_PVP01_0948800-E2	1083	185	17.082	2937.050	33.528	2.309	0.706	3.271	1.07E-03	1.40E-02	PVP01_0948800	TRAG16	tryptophan-rich protein
exon_PVP01_1340200-E1	927	84	9.061	1558.001	15.337	2.630	0.806	3.265	1.09E-03	1.43E-02	PVP01_1340200		conserved Plasmodium protein, unknown function
exon_PVP01_0904700-E1	186	76	40.860	7025.363	13.514	2.246	0.688	3.262	1.10E-03	1.44E-02	PVP01_0904700		conserved Plasmodium protein, unknown function
exon_PVP01_1304700-E8	1005	153	15.224	2617.541	27.661	2.373	0.728	3.261	1.11E-03	1.44E-02	PVP01_1304700		tetratricopeptide repeat protein, putative
exon_PVP01_1334600-E1	660	1943	294.394	50617.070	277.945	-1.557	0.477	-3.261	1.11E-03	1.44E-02	PVP01_1334600		60S ribosomal protein L10, putative
exon_PVP01_1233100-E1	1629	156	9.576	1646.538	26.939	1.951	0.599	3.259	1.12E-03	1.44E-02	PVP01_1233100		conserved Plasmodium protein, unknown function
exon_PVP01_0210200-E1	849	313	36.867	6338.767	45.998	-2.339	0.718	-3.256	1.13E-03	1.45E-02	PVP01_0210200		conserved protein, unknown function
exon_PVP01_0611600-E1	1986	93	4.683	805.141	16.215	2.343	0.720	3.255	1.13E-03	1.45E-02	PVP01_0611600	CDC20	cell division cycle protein 20 homolog, putative
exon_PVP01_1214400-E2	1134	53	4.674	803.583	9.178	2.586	0.794	3.256	1.13E-03	1.45E-02	PVP01_1214400		conserved Plasmodium protein, unknown function
exon_PVP01_1413700-E2	6405	156	2.436	418.768	27.034	2.054	0.631	3.257	1.13E-03	1.45E-02	PVP01_1413700		conserved Plasmodium protein, unknown function
exon_PVP01_1134500-E1	1764	218	12.358	2124.839	36.274	1.756	0.541	3.248	1.16E-03	1.48E-02	PVP01_1134500		conserved Plasmodium protein, unknown function
exon_PVP01_0312600-E3	984	747	75.915	13052.498	110.466	-1.658	0.511	-3.246	1.17E-03	1.49E-02	PVP01_0312600	EIF3I	eukaryotic translation initiation factor 3 subunit I, putative
exon_PVP01_1125600-E1	867	186	21.453	3688.604	32.866	1.940	0.598	3.243	1.18E-03	1.50E-02	PVP01_1125600		ribonuclease H2 subunit A, putative
exon_PVP01_0704000-E1	3354	161	4.800	825.336	28.935	2.188	0.675	3.240	1.20E-03	1.51E-02	PVP01_0704000	WDR66	WD repeat-containing protein 66, putative
exon_PVP01_0826600-E6	1917	114	5.947	1022.471	20.171	2.272	0.702	3.238	1.20E-03	1.52E-02	PVP01_0826600		conserved Plasmodium protein, unknown function
exon_PVP01_1321400-E1	846	1013	119.740	20587.671	172.789	1.588	0.491	3.234	1.22E-03	1.53E-02	PVP01_1321400		leucine-rich repeat protein
exon_PVP01_1264100-E1	9060	148	1.634	280.868	22.356	-2.114	0.655	-3.229	1.24E-03	1.56E-02	PVP01_1264100	SEC7	protein transport protein SEC7, putative
exon_PVP01_0206100-E1	819	195	23.810	4093.727	31.913	1.898	0.588	3.227	1.25E-03	1.56E-02	PVP01_0206100	PHIL1	photosensitized INA-labeled protein PHIL1, putative
exon_PVP01_1023600-E1	1299	191	14.704	2528.089	29.828	-2.352	0.729	-3.225	1.26E-03	1.56E-02	PVP01_1023600		PDCD2 domain-containing protein, putative
exon_PVP01_1143200-E1	798	47	5.890	1012.659	8.409	2.570	0.797	3.226	1.26E-03	1.56E-02	PVP01_1143200		conserved Plasmodium protein, unknown function
exon_PVP01_1260900-E1	2151	43	1.999	343.713	7.948	2.715	0.842	3.224	1.26E-03	1.56E-02	PVP01_1260900		conserved Plasmodium protein, unknown function
exon_PVP01_0611300-E5	537	57	10.615	1825.025	9.786	2.550	0.792	3.221	1.28E-03	1.58E-02	PVP01_0611300		conserved Plasmodium protein, unknown function
exon_PVP01_1011300-E1	1470	190	12.925	2222.309	31.563	2.049	0.636	3.219	1.29E-03	1.58E-02	PVP01_1011300		inner membrane complex protein, putative
exon_PVP01_1024300-E1	1191	54	4.534	779.561	9.684	2.575	0.800	3.219	1.29E-03	1.58E-02	PVP01_1024300		conserved Plasmodium protein, unknown function
exon_PVP01_0709400-E1	1644	456	27.737	4769.042	77.615	1.803	0.561	3.217	1.30E-03	1.58E-02	PVP01_0709400		DNA primase large subunit, putative
exon_PVP01_0920600-E1	2541	169	6.651	1143.537	30.299	2.220	0.690	3.217	1.30E-03	1.58E-02	PVP01_0920600		conserved protein, unknown function
exon_PVP01_1243100-E1	981	94	9.582	1647.506	14.355	-2.467	0.768	-3.213	1.31E-03	1.60E-02	PVP01_1243100	CYC1	cyclin 1, putative
exon_PVP01_1408700-E1	1329	364	27.389	4709.172	53.419	-1.934	0.602	-3.211	1.32E-03	1.60E-02	PVP01_1408700		FHA domain-containing protein, putative
exon_PVP01_0907600-E1	4029	204	5.063	870.565	30.173	-1.866	0.582	-3.205	1.35E-03	1.64E-02	PVP01_0907600		protein kinase, putative
exon_PVP01_0813100-E4	1590	149	9.371	1611.229	25.818	1.924	0.603	3.190	1.42E-03	1.72E-02	PVP01_0813100		zinc finger protein, putative
exon_PVP01_0409000-E1	4794	85	1.773	304.852	14.872	2.094	0.657	3.189	1.43E-03	1.72E-02	PVP01_0409000		DEAD/DEAH box helicase, putative
exon_PVP01_0618900-E1	1440	129	8.958	1540.265	22.743	2.118	0.665	3.184	1.45E-03	1.74E-02	PVP01_0618900		aspartyl protease, putative
exon_PVP01_0117300-E1	411	173	42.092	7237.231	29.597	1.717	0.540	3.182	1.46E-03	1.75E-02	PVP01_0117300		conserved Plasmodium protein, unknown function
exon_PVP01_0506400-E1	807	90	11.152	1917.508	15.370	2.112	0.664	3.178	1.48E-03	1.77E-02	PVP01_0506400		rhomboid protease ROM3, putative
exon_PVP01_0616200-E2	579	81	13.990	2405.330	11.136	-2.685	0.845	-3.177	1.49E-03	1.77E-02	PVP01_0616200		conserved Plasmodium protein, unknown function
exon_PVP01_1026600-E1	450	200	44.444	7641.623	34.569	1.729	0.545	3.174	1.50E-03	1.78E-02	PVP01_1026600		conserved Plasmodium protein, unknown function
exon_PVP01_1236900-E2	1290	85	6.589	1132.915	15.728	2.364	0.745	3.175	1.50E-03	1.78E-02	PVP01_1236900		protein MGET, putative
exon_PVP01_1448000-E2	5619	58	1.032	177.475	10.285	2.486	0.784	3.173	1.51E-03	1.78E-02	PVP01_1448000	MyoJ	myosin J, putative
exon_PVP01_0622700-E4	1098	45	4.098	704.658	8.205	2.596	0.819	3.168	1.53E-03	1.81E-02	PVP01_0622700		conserved Plasmodium protein, unknown function
exon_PVP01_0709200-E1	1800	112	6.222	1069.827	19.600	1.982	0.626	3.166	1.55E-03	1.82E-02	PVP01_0709200	ALP5a	actin-like protein, putative
exon_PVP01_0605400-E2	2658	30	1.129	194.059	5.442	2.865	0.907	3.158	1.59E-03	1.86E-02	PVP01_0605400		conserved Plasmodium protein, unknown function
exon_PVP01_0720800-E1	978	85	8.691	1494.336	14.707	2.198	0.696	3.155	1.60E-03	1.87E-02	PVP01_0720800		conserved protein, unknown function
exon_PVP01_1215900-E7	1698	61	3.592	617.675	10.856	2.612	0.828	3.154	1.61E-03	1.88E-02	PVP01_1215900		CPW-WPC family protein
exon_PVP01_0105600-E1	2499	316	12.645	2174.147	123.394	1.719	0.546	3.147	1.65E-03	1.92E-02	PVP01_0105600	MCM7	DNA replication licensing factor MCM7, putative
exon_PVP01_0702200-E2	4275	223	5.216	896.885	38.493	1.952	0.621	3.142	1.68E-03	1.94E-02	PVP01_0702200	CCp4	LCCL domain-containing protein
exon_PVP01_1301500-E1	972	588	60.494	10401.098	99.644	1.593	0.507	3.142	1.68E-03	1.94E-02	PVP01_1301500	MPC	mitochondrial phosphate carrier protein, putative
exon_PVP01_0703200-E2	1458	652	44.719	7688.794	112.232	1.845	0.588	3.141	1.69E-03	1.94E-02	PVP01_0703200	RPA1	replication protein A1, small fragment, putative
exon_PVP01_1259500-E2	441	47	10.658	1832.430	8.027	2.513	0.803	3.131	1.74E-03	2.00E-02	PVP01_1259500		conserved Plasmodium protein, unknown function
exon_PVP01_0941800-E1	7392	121	1.637	281.444	21.454	2.099	0.671	3.130	1.75E-03	2.01E-02	PVP01_0941800		conserved Plasmodium protein, unknown function

↳

Exon ID	TL	Reads count	CVGE	RPKM	Mean counts	Log2FC	SD	Wald stat	p-value	q-value	Gene ID	Symbol	Product description
exon_PVP01_0506800-E1	4998	155	3.101	533.217	23.440	-2.065	0.660	-3.128	1.76E-03	2.02E-02	PVP01_0506800		conserved Plasmodium protein, unknown function
exon_PVP01_1307900-E1	2820	217	7.695	1323.058	37.747	1.808	0.580	3.115	1.84E-03	2.10E-02	PVP01_1307900		zinc finger protein, putative
exon_PVP01_1346900-E1	1245	46	3.695	635.267	8.300	2.638	0.847	3.114	1.85E-03	2.10E-02	PVP01_1346900		conserved protein, unknown function
exon_PVP01_0507000-E1	636	283	44.497	7650.635	49.329	2.002	0.644	3.110	1.87E-03	2.12E-02	PVP01_0507000		conserved Plasmodium protein, unknown function
exon_PVP01_0514500-E4	4410	68	1.542	265.118	12.153	2.370	0.762	3.111	1.87E-03	2.12E-02	PVP01_0514500		conserved Plasmodium protein, unknown function
exon_PVP01_0114700-E1	1299	44	3.387	582.387	7.896	2.446	0.787	3.107	1.89E-03	2.14E-02	PVP01_0114700		conserved Plasmodium protein, unknown function
exon_PVP01_0724800-E1	2853	181	6.344	1090.800	32.145	2.029	0.654	3.105	1.91E-03	2.15E-02	PVP01_0724800		protein kinase, putative
exon_PVP01_1317600-E1	2322	95	4.091	703.444	16.409	2.158	0.696	3.102	1.92E-03	2.16E-02	PVP01_1317600	ISC3	inner membrane complex suture component, putative
exon_PVP01_1008300-E1	4860	48	0.988	169.814	7.672	-3.005	0.970	-3.100	1.94E-03	2.17E-02	PVP01_1008300		WD repeat-containing protein, putative
exon_PVP01_1122700-E3	1536	326	21.224	3649.174	44.926	-1.883	0.608	-3.099	1.94E-03	2.17E-02	PVP01_1122700		pyruvate kinase, putative
exon_PVP01_1143600-E4	1065	245	23.005	3955.347	41.301	1.943	0.627	3.098	1.95E-03	2.17E-02	PVP01_1143600	ARK1	serine/threonine protein kinase, putative
exon_PVP01_1433300-E6	564	31	5.496	945.041	5.350	2.788	0.900	3.097	1.96E-03	2.18E-02	PVP01_1433300		conserved Plasmodium protein, unknown function
exon_PVP01_1206400-E1	10353	134	1.294	222.539	22.471	1.842	0.595	3.095	1.97E-03	2.19E-02	PVP01_1206400		conserved Plasmodium protein, unknown function
exon_PVP01_0923500-E1	16086	147	0.914	157.122	26.014	1.927	0.623	3.093	1.98E-03	2.20E-02	PVP01_0923500		dynein heavy chain, putative
exon_PVP01_0611400-E1	1716	171	9.965	1713.353	28.817	1.790	0.580	3.086	2.03E-03	2.24E-02	PVP01_0611400		conserved protein, unknown function
exon_PVP01_1269700-E3	810	472	58.272	10019.017	69.741	-1.624	0.526	-3.085	2.03E-03	2.24E-02	PVP01_1269700		proteasome subunit alpha type-1, putative
exon_PVP01_1027600-E5	381	72	18.898	3249.194	12.359	2.218	0.719	3.083	2.05E-03	2.25E-02	PVP01_1027600		conserved Plasmodium protein, unknown function
exon_PVP01_0506400-E4	807	43	5.328	916.143	7.392	2.593	0.841	3.082	2.05E-03	2.25E-02	PVP01_0506400		rhomboid protease ROM3, putative
exon_PVP01_1140600-E1	1416	239	16.879	2902.036	34.001	-1.771	0.575	-3.082	2.06E-03	2.25E-02	PVP01_1140600	SAM50	sorting assembly machinery 50 kDa subunit, putative
exon_PVP01_0409400-E1	1095	178	16.256	2794.950	29.046	1.816	0.590	3.077	2.09E-03	2.28E-02	PVP01_0409400		conserved Plasmodium protein, unknown function
exon_PVP01_1235100-E1	2439	27	1.107	190.336	4.629	2.998	0.975	3.076	2.10E-03	2.28E-02	PVP01_1235100		conserved Plasmodium protein, unknown function
exon_PVP01_1404800-E5	612	42	6.863	1179.957	7.698	2.740	0.893	3.069	2.15E-03	2.33E-02	PVP01_1404800		conserved protein, unknown function
exon_PVP01_0723100-E1	5874	149	2.537	436.135	25.262	2.025	0.660	3.067	2.16E-03	2.34E-02	PVP01_0723100		conserved Plasmodium protein, unknown function
exon_PVP01_0108700-E2	2247	313	13.930	2395.021	47.922	-1.635	0.534	-3.062	2.20E-03	2.37E-02	PVP01_0108700		heat shock protein 90, putative
exon_PVP01_1119500-E1	849	45	5.300	911.324	7.940	2.551	0.833	3.061	2.21E-03	2.37E-02	PVP01_1119500		CPW-WPC family protein
exon_PVP01_1444800-E2	795	536	67.421	11592.198	89.581	1.486	0.485	3.061	2.21E-03	2.37E-02	PVP01_1444800	PCNA2	proliferating cell nuclear antigen 2, putative
exon_PVP01_1130000-E1	3945	219	5.551	954.477	32.969	-1.919	0.628	-3.057	2.24E-03	2.40E-02	PVP01_1130000	ACS12	acyl-CoA synthetase, putative
exon_PVP01_1314600-E1	1011	72	7.122	1224.474	12.535	2.066	0.676	3.055	2.25E-03	2.41E-02	PVP01_1314600		conserved Plasmodium protein, unknown function
exon_PVP01_1106400-E1	1629	54	3.315	569.955	9.244	2.386	0.782	3.052	2.27E-03	2.42E-02	PVP01_1106400	TOP6B	DNA topoisomerase 6 subunit B, putative
exon_PVP01_0725900-E5	2568	64	2.492	428.502	11.236	2.197	0.720	3.050	2.29E-03	2.44E-02	PVP01_0725900		conserved Plasmodium protein, unknown function
exon_PVP01_1207200-E14	834	47	5.635	968.947	8.367	2.418	0.794	3.044	2.33E-03	2.48E-02	PVP01_1207200		conserved protein, unknown function
exon_PVP01_1206000-E1	5010	90	1.796	308.868	15.820	2.227	0.734	3.036	2.40E-03	2.53E-02	PVP01_1206000		E1-E2 ATPase, putative
exon_PVP01_1314400-E1	1035	510	49.275	8472.234	91.264	1.846	0.608	3.036	2.40E-03	2.53E-02	PVP01_1314400		ribonuclease H2 subunit B, putative
exon_PVP01_1458800-E2	1011	851	84.174	14472.599	146.479	1.719	0.567	3.033	2.42E-03	2.56E-02	PVP01_1458800	RFC4	replication factor C subunit 4, putative
exon_PVP01_0527300-E1	2148	203	9.451	1624.912	36.526	1.931	0.637	3.030	2.45E-03	2.57E-02	PVP01_0527300		conserved Plasmodium protein, unknown function
exon_PVP01_1145900-E1	1392	160	11.494	1976.282	26.559	1.708	0.564	3.030	2.44E-03	2.57E-02	PVP01_1145900		conserved Plasmodium protein, unknown function
exon_PVP01_0921300-E1	3603	117	3.247	558.328	20.749	2.022	0.668	3.028	2.46E-03	2.58E-02	PVP01_0921300		conserved Plasmodium protein, unknown function
exon_PVP01_0530300-E1	411	151	36.740	6316.889	26.982	1.955	0.646	3.027	2.47E-03	2.58E-02	PVP01_0530300		conserved Plasmodium protein, unknown function
exon_PVP01_0602200-E1	3291	345	10.483	1802.434	59.931	1.611	0.532	3.026	2.48E-03	2.58E-02	PVP01_0602200		DNA polymerase delta catalytic subunit, putative
exon_PVP01_0721000-E1	1251	1285	102.718	17660.946	193.967	-1.381	0.456	-3.025	2.49E-03	2.58E-02	PVP01_0721000	PGK	phosphoglycerate kinase, putative
exon_PVP01_0730300-E1	765	63	8.235	1415.948	11.531	2.468	0.816	3.026	2.48E-03	2.58E-02	PVP01_0730300		conserved protein, unknown function
exon_PVP01_1443200-E1	4914	91	1.852	318.401	13.527	-2.367	0.784	-3.019	2.54E-03	2.63E-02	PVP01_1443200		conserved Plasmodium protein, unknown function
exon_PVP01_0934000-E1	4239	163	3.845	661.138	20.419	-2.449	0.812	-3.016	2.56E-03	2.64E-02	PVP01_0934000		conserved Plasmodium protein, unknown function
exon_PVP01_0518800-E2	516	57	11.047	1899.299	10.044	2.274	0.755	3.013	2.58E-03	2.66E-02	PVP01_0518800	HSP20	small heat shock protein HSP20, putative
exon_PVP01_0512700-E1	1677	184	10.972	1886.483	26.622	-2.110	0.702	-3.007	2.64E-03	2.71E-02	PVP01_0512700	CLP1	polyribonucleotide 5'-hydroxyl-kinase Clp1, putative
exon_PVP01_0823400-E2	684	131	19.152	3292.936	17.113	-2.265	0.753	-3.007	2.64E-03	2.71E-02	PVP01_0823400	YOP1	protein YOP1, putative
exon_PVP01_0320200-E3	915	27	2.951	507.354	4.782	2.788	0.928	3.004	2.66E-03	2.72E-02	PVP01_0320200		conserved Plasmodium protein, unknown function
exon_PVP01_1138700-E1	411	1248	303.650	52208.462	203.300	1.411	0.470	3.004	2.66E-03	2.72E-02	PVP01_1138700	H3	histone H3, putative
exon_PVP01_1209900-E1	4752	136	2.862	492.074	23.603	1.946	0.648	3.003	2.68E-03	2.72E-02	PVP01_1209900		conserved Plasmodium protein, unknown function
exon_PVP01_1212200-E3	2454	455	18.541	3187.902	79.526	1.742	0.580	3.002	2.68E-03	2.72E-02	PVP01_1212200		myosin A, putative
exon_PVP01_0510900-E2	1539	46	2.989	513.910	8.353	2.464	0.821	3.001	2.69E-03	2.73E-02	PVP01_0510900		alpha/beta hydrolase, putative
exon_PVP01_1238800-E1	5700	34	0.596	102.559	6.299	2.780	0.927	2.999	2.71E-03	2.74E-02	PVP01_1238800		conserved Plasmodium protein, unknown function
exon_PVP01_0411100-E1	2148	69	3.212	552.310	12.131	2.055	0.686	2.996	2.74E-03	2.76E-02	PVP01_0411100		conserved protein, unknown function
exon_PVP01_1449500-E1	2880	42	1.458	250.741	7.554	2.489	0.831	2.995	2.74E-03	2.76E-02	PVP01_1449500		conserved Plasmodium protein, unknown function
exon_PVP01_0722000-E1	834	102	12.230	2102.821	16.961	2.147	0.717	2.995	2.75E-03	2.76E-02	PVP01_0722000		cyclin-dependent kinases regulatory subunit, putative
exon_PVP01_1135500-E2	2715	104	3.831	658.615	17.752	2.023	0.680	2.977	2.92E-03	2.92E-02	PVP01_1135500		conserved Plasmodium protein, unknown function
exon_PVP01_0419800-E1	2034	185	9.095	1563.828	34.011	2.049	0.689	2.974	2.94E-03	2.94E-02	PVP01_0419800		conserved Plasmodium protein, unknown function
exon_PVP01_0817600-E1	4209	111	2.637	453.432	20.311	2.152	0.724	2.971	2.97E-03	2.96E-02	PVP01_0817600		conserved Plasmodium protein, unknown function
exon_PVP01_1243800-E1	969	179	18.473	3176.124	30.685	1.710	0.576	2.971	2.97E-03	2.96E-02	PVP01_1243800	MED20	mediator of RNA polymerase II transcription subunit 20, putative
exon_PVP01_1306000-E4	2610	131	5.019	862.976	22.999	2.011	0.678	2.966	3.02E-03	3.00E-02	PVP01_1306000		conserved Plasmodium protein, unknown function
exon_PVP01_1454700-E2	459	46	10.022	1723.111	8.316	2.433	0.822	2.961	3.07E-03	3.04E-02	PVP01_1454700		p25-alpha family protein, putative
exon_PVP01_1117900-E1	741	291	39.271	6752.163	40.967	-1.852	0.626	-2.959	3.09E-03	3.05E-02	PVP01_1117900		proteasome subunit alpha type-4, putative
exon_PVP01_0728000-E1	627	74	11.802	2029.235	13.500	2.300	0.778	2.956	3.12E-03	3.07E-02	PVP01_0728000		conserved protein, unknown function
exon_PVP01_1441000-E7	789	48	6.084	1046.002	8.489	2.340	0.792	2.956	3.12E-03	3.07E-02	PVP01_1441000		conserved Plasmodium protein, unknown function
exon_PVP01_0803400-E1	861	271	31.475	5411.707	46.442	1.886	0.640	2.950	3.18E-03	3.12E-02	PVP01_0803400	IMC1c	inner membrane complex protein 1c, putative

Exon ID	TL	Reads count	CVGE	RPKM	Mean counts	Log2FC	SD	Wald stat	p-value	q-value	Gene ID	Symbol	Product description
exon_PVP01_0806000-E1	396	64	16.162	2778.772	11.272	2.325	0.788	2.949	3.19E-03	3.12E-02	PVP01_0806000		conserved Plasmodium protein, unknown function
exon_PVP01_1327600-E5	2682	59	2.200	378.235	10.818	2.476	0.839	2.950	3.18E-03	3.12E-02	PVP01_1327600		conserved Plasmodium protein, unknown function
exon_PVP01_0913400-E1	2034	377	18.535	3186.827	63.910	1.667	0.566	2.947	3.20E-03	3.13E-02	PVP01_0913400		ATPase, putative
exon_PVP01_1422600-E1	2889	134	4.638	797.490	25.045	2.309	0.784	2.944	3.24E-03	3.16E-02	PVP01_1422600		conserved Plasmodium protein, unknown function
exon_PVP01_1205900-E1	360	121	33.611	5778.978	17.710	-2.171	0.738	-2.941	3.28E-03	3.18E-02	PVP01_1205900		conserved Plasmodium protein, unknown function
exon_PVP01_0111600-E1	2595	114	4.393	755.328	19.558	1.790	0.609	2.937	3.31E-03	3.20E-02	PVP01_0111600		conserved Plasmodium protein, unknown function
exon_PVP01_0938600-E2	1467	115	7.839	1347.832	20.565	1.967	0.669	2.937	3.31E-03	3.20E-02	PVP01_0938600		conserved Plasmodium protein, unknown function
exon_PVP01_1208800-E1	2100	41	1.952	335.686	6.962	2.423	0.826	2.933	3.36E-03	3.24E-02	PVP01_1208800		dynactin subunit 2, putative
exon_PVP01_0836300-E2	588	106	18.027	3099.536	18.467	2.063	0.704	2.932	3.37E-03	3.24E-02	PVP01_0836300		phosphatidylethanolamine-binding protein, putative
exon_PVP01_1204500-E1	1023	381	37.243	6403.501	56.352	-1.498	0.511	-2.931	3.38E-03	3.25E-02	PVP01_1204500		conserved protein, unknown function
exon_PVP01_1459500-E3	1362	56	4.112	706.934	9.860	2.226	0.761	2.925	3.45E-03	3.31E-02	PVP01_1459500		GAS8-like protein, putative
exon_PVP01_1033100-E2	1146	149	13.002	2235.475	20.821	-2.122	0.726	-2.924	3.46E-03	3.31E-02	PVP01_1033100		conserved protein, unknown function
exon_PVP01_0205700-E2	507	291	57.396	9868.546	48.602	1.615	0.553	2.922	3.47E-03	3.32E-02	PVP01_0205700		tubulin-specific chaperone a, putative
exon_PVP01_0421100-E1	2391	55	2.300	395.504	10.374	2.467	0.845	2.920	3.50E-03	3.34E-02	PVP01_0421100		condensin-2 complex subunit H2, putative
exon_PVP01_0833000-E3	771	1382	179.248	30819.231	198.842	-1.521	0.521	-2.919	3.51E-03	3.34E-02	PVP01_0833000		60S ribosomal protein L7, putative
exon_PVP01_0710000-E1	654	91	13.914	2392.389	15.844	2.096	0.718	2.918	3.53E-03	3.35E-02	PVP01_0710000		conserved Plasmodium protein, unknown function
exon_PVP01_0611900-E2	792	823	103.914	17866.636	119.209	-1.552	0.533	-2.915	3.56E-03	3.37E-02	PVP01_0611900	RPS2	40S ribosomal protein S2, putative
exon_PVP01_1321300-E1	1248	131	10.497	1804.782	22.060	1.746	0.599	2.914	3.57E-03	3.37E-02	PVP01_1321300		conserved Plasmodium protein, unknown function
exon_PVP01_0207800-E1	507	170	33.531	5765.130	28.087	1.632	0.561	2.909	3.62E-03	3.41E-02	PVP01_0207800	CEN1	centrin-1, putative
exon_PVP01_0938900-E1	2406	88	3.658	628.862	15.462	2.278	0.783	2.909	3.63E-03	3.41E-02	PVP01_0938900		conserved Plasmodium protein, unknown function
exon_PVP01_0111000-E1	453	292	64.459	11082.884	41.613	-1.616	0.556	-2.905	3.67E-03	3.44E-02	PVP01_0111000	RPL34	60S ribosomal protein L34, putative
exon_PVP01_0930200-E1	1842	93	5.049	868.083	13.781	-2.196	0.756	-2.905	3.68E-03	3.44E-02	PVP01_0930200		A/G-specific adenine glycosylase, putative
exon_PVP01_1019400-E6	651	29	4.455	765.923	5.256	2.599	0.896	2.902	3.70E-03	3.46E-02	PVP01_1019400		conserved Plasmodium protein, unknown function
exon_PVP01_1139200-E4	855	27	3.158	542.957	4.712	2.748	0.947	2.901	3.73E-03	3.47E-02	PVP01_1139200	DHHC2	palmitoyltransferase DHHC2, putative
exon_PVP01_1341100-E1	6195	228	3.680	632.793	37.161	1.537	0.531	2.897	3.77E-03	3.51E-02	PVP01_1341100		conserved Plasmodium protein, unknown function
exon_PVP01_0830700-E2	1842	115	6.243	1073.436	15.905	-2.196	0.760	-2.891	3.84E-03	3.57E-02	PVP01_0830700	AS	asparagine synthetase [glutamine-hydrolyzing], putative
exon_PVP01_0704900-E1	2433	78	3.206	551.214	13.935	2.107	0.729	2.890	3.85E-03	3.57E-02	PVP01_0704900		dynein intermediate light chain, putative
exon_PVP01_1342300-E1	1827	139	7.608	1308.110	21.499	-1.930	0.668	-2.887	3.89E-03	3.59E-02	PVP01_1342300	PPR	pentatricopeptide repeat domain-containing protein, putative
exon_PVP01_0823100-E2	762	171	22.441	3858.418	23.542	-1.860	0.645	-2.886	3.90E-03	3.60E-02	PVP01_0823100		proteasome subunit alpha type-3, putative
exon_PVP01_1245100-E1	402	32	7.960	1368.649	5.584	2.563	0.889	2.884	3.93E-03	3.62E-02	PVP01_1245100		conserved Plasmodium protein, unknown function
exon_PVP01_0731000-E1	1680	287	17.083	2937.249	42.267	-1.931	0.671	-2.879	3.99E-03	3.67E-02	PVP01_0731000	M18AAP	M18 aspartyl aminopeptidase, putative
exon_PVP01_0907000-E1	1977	172	8.700	1495.856	25.933	-1.811	0.630	-2.875	4.04E-03	3.71E-02	PVP01_0907000		conserved Plasmodium protein, unknown function
exon_PVP01_0934500-E1	2277	42	1.845	317.142	6.587	-2.820	0.981	-2.874	4.05E-03	3.71E-02	PVP01_0934500		FHA domain-containing protein, putative
exon_PVP01_1414600-E1	831	167	20.096	3455.283	24.064	-2.063	0.719	-2.868	4.13E-03	3.77E-02	PVP01_1414600		Maf-like protein, putative
exon_PVP01_1145300-E2	6984	46	0.659	113.246	7.834	2.314	0.808	2.863	4.19E-03	3.82E-02	PVP01_1145300		conserved Plasmodium protein, unknown function
exon_PVP01_0211800-E1	1896	326	17.194	2956.293	47.946	-1.643	0.574	-2.862	4.21E-03	3.83E-02	PVP01_0211800		aspartate--tRNA ligase, putative
exon_PVP01_1017100-E2	783	933	119.157	20487.455	135.504	-1.372	0.480	-2.855	4.30E-03	3.90E-02	PVP01_1017100	RPL2	60S ribosomal protein L2, putative
exon_PVP01_1433900-E1	2781	97	3.488	599.707	17.689	1.963	0.688	2.854	4.31E-03	3.91E-02	PVP01_1433900		SUN domain-containing protein, putative
exon_PVP01_0203900-E1	3318	190	5.726	984.567	30.871	1.602	0.562	2.852	4.35E-03	3.93E-02	PVP01_0203900		P-loop containing nucleoside triphosphate hydrolase, putative
exon_PVP01_1119000-E1	1542	54	3.502	602.112	8.307	-2.623	0.920	-2.851	4.36E-03	3.94E-02	PVP01_1119000	YTM1	ribosome biogenesis protein YTM1, putative
exon_PVP01_1214900-E1	2460	83	3.374	580.111	14.414	1.902	0.667	2.850	4.37E-03	3.94E-02	PVP01_1214900	ABCB5	ABC transporter B family member 5, putative
exon_PVP01_1415500-E1	1272	153	12.028	2068.104	23.228	-1.832	0.643	-2.849	4.38E-03	3.94E-02	PVP01_1415500	LipL1	lipoate-protein ligase 1, putative
exon_PVP01_0602900-E1	1572	133	8.461	1454.679	23.212	1.894	0.666	2.845	4.44E-03	3.98E-02	PVP01_0602900		conserved Plasmodium protein, unknown function
exon_PVP01_1436200-E1	2892	46	1.591	273.481	7.921	2.297	0.808	2.844	4.46E-03	3.99E-02	PVP01_1436200		conserved protein, unknown function
exon_PVP01_1137000-E1	1863	400	21.471	3691.605	58.969	-1.473	0.519	-2.839	4.53E-03	4.04E-02	PVP01_1137000	EIF3L	eukaryotic translation initiation factor 3 subunit L, putative
exon_PVP01_1250500-E1	2784	29	1.042	179.101	5.354	2.544	0.896	2.838	4.54E-03	4.05E-02	PVP01_1250500		conserved Plasmodium protein, unknown function
exon_PVP01_0941100-E2	498	49	9.839	1691.745	8.565	2.275	0.802	2.836	4.57E-03	4.06E-02	PVP01_0941100		conserved Plasmodium protein, unknown function
exon_PVP01_0705200-E1	1245	291	23.373	4018.757	44.354	-1.879	0.663	-2.833	4.61E-03	4.09E-02	PVP01_0705200	LRR9	leucine-rich repeat protein
exon_PVP01_1228100-E2	1842	91	4.940	849.415	16.082	2.114	0.746	2.833	4.61E-03	4.09E-02	PVP01_1228100		RNA-binding protein, putative
exon_PVP01_0933800-E1	90	153	170.000	29229.209	25.074	1.686	0.596	2.832	4.63E-03	4.10E-02	PVP01_0933800		conserved Plasmodium protein, unknown function
exon_PVP01_1448500-E1	855	186	21.754	3740.373	32.597	2.039	0.720	2.831	4.64E-03	4.10E-02	PVP01_1448500	SPM2	subpellicular microtubule protein 2, putative
exon_PVP01_0206000-E2	3270	423	12.936	2224.133	69.721	1.609	0.569	2.829	4.67E-03	4.12E-02	PVP01_0206000	CCp5	LCCL domain-containing protein, putative
exon_PVP01_0811100-E1	435	28	6.437	1106.718	5.111	2.668	0.944	2.826	4.72E-03	4.13E-02	PVP01_0811100	ISP1	inner membrane complex sub-compartment protein 1, putative
exon_PVP01_0921600-E1	870	80	9.195	1581.025	13.896	2.031	0.719	2.825	4.72E-03	4.13E-02	PVP01_0921600	DHHC3	palmitoyltransferase DHHC3, putative
exon_PVP01_0928000-E1	861	226	26.249	4513.084	34.333	-1.762	0.624	-2.824	4.75E-03	4.13E-02	PVP01_0928000		tRNA (guanine-N(7)-)-methyltransferase, putative
exon_PVP01_1019200-E1	4131	93	2.251	387.076	15.975	2.317	0.820	2.824	4.75E-03	4.13E-02	PVP01_1019200		MORN repeat protein, putative
exon_PVP01_1325400-E2	855	36	4.211	723.943	6.299	2.433	0.861	2.825	4.73E-03	4.13E-02	PVP01_1325400		calcium-dependent protein kinase, putative
exon_PVP01_1332400-E1	1413	152	10.757	1849.565	25.136	1.608	0.570	2.824	4.75E-03	4.13E-02	PVP01_1332400	GDH1	NADP-specific glutamate dehydrogenase, putative
exon_PVP01_0318900-E4	1209	91	7.527	1294.146	15.742	1.975	0.700	2.822	4.77E-03	4.14E-02	PVP01_0318900		conserved Plasmodium protein, unknown function
exon_PVP01_1022900-E1	816	49	6.005	1032.462	8.554	2.224	0.790	2.817	4.85E-03	4.21E-02	PVP01_1022900		conserved Plasmodium protein, unknown function
exon_PVP01_1312800-E1	2586	77	2.978	511.953	12.098	-2.215	0.787	-2.816	4.87E-03	4.21E-02	PVP01_1312800		conserved protein, unknown function
exon_PVP01_1109400-E1	795	66	8.302	1427.398	11.620	2.067	0.734	2.815	4.88E-03	4.22E-02	PVP01_1109400		conserved protein, unknown function
exon_PVP01_1025600-E2	1107	92	8.311	1428.921	16.578	2.158	0.767	2.814	4.89E-03	4.22E-02	PVP01_1025600		conserved protein, unknown function
exon_PVP01_1255400-E1	2625	27	1.029	176.849	4.635	2.592	0.922	2.811	4.93E-03	4.24E-02	PVP01_1255400		LCCL domain-containing protein, putative
exon_PVP01_0207200-E1	1533	29	1.892	325.255	4.670	-3.058	1.090	-2.806	5.01E-03	4.29E-02	PVP01_0207200		dolichyl-diphosphooligosaccharide--protein glycosyltransferase subunit OST3/OST6, putative

Exon ID	TL	Reads count	CVGE	RPKM	Mean counts	Log2FC	SD	Wald stat	p-value	q-value	Gene ID	Symbol	Product description
exon_PVP01_1029500-E5	831	33	3.971	682.780	5.812	2.414	0.860	2.806	5.01E-03	4.29E-02	PVP01_1029500		MOLO1 domain-containing protein, putative
exon_PVP01_1031800-E1	2604	101	3.879	666.881	17.819	1.914	0.684	2.798	5.14E-03	4.39E-02	PVP01_1031800		conserved Plasmodium protein, unknown function
exon_PVP01_1413500-E1	204	65	31.863	5478.370	11.336	2.198	0.786	2.798	5.14E-03	4.39E-02	PVP01_1413500		conserved Plasmodium protein, unknown function
exon_PVP01_0826600-E5	1917	33	1.721	295.978	5.926	2.601	0.931	2.795	5.19E-03	4.42E-02	PVP01_0826600		conserved Plasmodium protein, unknown function
exon_PVP01_0834500-E2	420	134	31.905	5485.594	18.806	-1.861	0.666	-2.795	5.20E-03	4.42E-02	PVP01_0834500		ubiquitin-conjugating enzyme E2, putative
exon_PVP01_1252200-E1	570	39	6.842	1176.408	6.840	2.373	0.850	2.793	5.22E-03	4.43E-02	PVP01_1252200		conserved Plasmodium protein, unknown function
exon_PVP01_1116300-E1	1929	96	4.977	855.672	16.275	1.935	0.693	2.792	5.24E-03	4.44E-02	PVP01_1116300	PIP1	PhIL1 interacting protein PIP1, putative
exon_PVP01_0106600-E1	279	1188	425.806	73211.680	168.135	-1.521	0.546	-2.789	5.29E-03	4.44E-02	PVP01_0106600	RPL37	60S ribosomal protein L37, putative
exon_PVP01_0205000-E1	684	33	4.825	829.518	5.980	2.388	0.858	2.785	5.36E-03	4.44E-02	PVP01_0205000		conserved Plasmodium protein, unknown function
exon_PVP01_0709900-E2	3738	39	1.043	179.388	6.109	-2.748	0.987	-2.784	5.37E-03	4.44E-02	PVP01_0709900		conserved Plasmodium protein, unknown function
exon_PVP01_0731800-E1	2145	47	2.191	376.737	8.439	2.364	0.849	2.786	5.34E-03	4.44E-02	PVP01_0731800		conserved Plasmodium protein, unknown function
exon_PVP01_0938500-E1	513	110	21.442	3686.748	15.657	-2.022	0.726	-2.786	5.33E-03	4.44E-02	PVP01_0938500		30S ribosomal protein S14, putative
exon_PVP01_1131200-E2	447	935	209.172	35964.351	140.955	-1.429	0.512	-2.789	5.28E-03	4.44E-02	PVP01_1131200		60S ribosomal protein L27a, putative
exon_PVP01_1210200-E1	1329	40	3.010	517.491	6.078	-2.749	0.987	-2.787	5.33E-03	4.44E-02	PVP01_1210200	LipA	lipoyl synthase, putative
exon_PVP01_1231500-E2	573	1456	254.101	43689.280	209.562	-1.481	0.532	-2.786	5.34E-03	4.44E-02	PVP01_1231500		60S ribosomal protein L6, putative
exon_PVP01_1245900-E1	825	43	5.212	896.154	6.018	-2.742	0.985	-2.785	5.36E-03	4.44E-02	PVP01_1245900		30S ribosomal protein S10, putative
exon_PVP01_1311000-E2	462	707	153.030	26311.498	113.259	1.237	0.444	2.785	5.35E-03	4.44E-02	PVP01_1311000		polyubiquitin, putative
exon_PVP01_1437600-E1	1053	43	4.084	702.115	7.610	2.349	0.842	2.790	5.27E-03	4.44E-02	PVP01_1437600		secreted ookinete protein, putative
exon_PVP01_1454300-E1	2604	59	2.266	389.564	9.916	2.010	0.722	2.786	5.33E-03	4.44E-02	PVP01_1454300		conserved Plasmodium protein, unknown function
exon_PVP01_1405000-E2	2802	124	4.425	760.890	21.526	1.914	0.689	2.780	5.43E-03	4.49E-02	PVP01_1405000	LigI	DNA ligase I, putative
exon_PVP01_0208600-E2	648	107	16.512	2839.075	17.295	1.792	0.645	2.776	5.50E-03	4.54E-02	PVP01_0208600		EF hand domain-containing protein, putative
exon_PVP01_0108300-E1	336	246	73.214	12588.210	39.891	1.569	0.566	2.774	5.54E-03	4.54E-02	PVP01_0108300		conserved Plasmodium protein, unknown function
exon_PVP01_0415900-E1	6648	161	2.422	416.393	29.037	1.929	0.695	2.774	5.53E-03	4.54E-02	PVP01_0415900	P230p	6-cysteine protein P230p, putative
exon_PVP01_1426300-E2	648	705	108.796	18706.057	100.292	-1.542	0.556	-2.775	5.52E-03	4.54E-02	PVP01_1426300		60S ribosomal protein L13-2, putative
exon_PVP01_0906000-E1	1407	38	2.701	464.363	6.855	2.310	0.835	2.768	5.64E-03	4.62E-02	PVP01_0906000	WDR8	WD repeat-containing protein WRAP73, putative
exon_PVP01_0943900-E1	966	62	6.418	1103.526	8.945	-2.375	0.859	-2.765	5.70E-03	4.66E-02	PVP01_0943900		DnaJ protein, putative
exon_PVP01_1232300-E3	4059	195	4.804	826.007	33.703	1.789	0.647	2.764	5.71E-03	4.66E-02	PVP01_1232300		conserved Plasmodium protein, unknown function
exon_PVP01_0820600-E3	471	242	51.380	8834.106	32.851	-1.712	0.620	-2.762	5.74E-03	4.67E-02	PVP01_0820600	EF-1delta	elongation factor 1-delta, putative
exon_PVP01_1305600-E1	1725	94	5.449	936.929	16.579	2.137	0.774	2.762	5.74E-03	4.67E-02	PVP01_1305600		Tat binding protein 1(TBP-1)-interacting protein, putative
exon_PVP01_1464100-E5	1089	104	9.550	1642.002	17.717	1.762	0.638	2.761	5.77E-03	4.68E-02	PVP01_1464100	DIA2	DNA replication origin binding protein, putative
exon_PVP01_1419600-E1	2475	73	2.949	507.126	12.686	1.993	0.723	2.757	5.84E-03	4.73E-02	PVP01_1419600		conserved protein, unknown function
exon_PVP01_1024300-E8	1191	79	6.633	1140.469	13.382	1.924	0.699	2.752	5.92E-03	4.78E-02	PVP01_1024300		conserved Plasmodium protein, unknown function
exon_PVP01_1137700-E1	1164	45	3.866	664.703	7.662	2.164	0.786	2.752	5.92E-03	4.78E-02	PVP01_1137700		conserved protein, unknown function
exon_PVP01_0905200-E1	1293	473	36.582	6289.712	66.825	-1.535	0.558	-2.750	5.96E-03	4.80E-02	PVP01_0905200	Trx-mero	thioredoxin-like mero protein, putative
exon_PVP01_1435500-E1	3315	117	3.529	606.835	19.910	1.662	0.605	2.750	5.97E-03	4.80E-02	PVP01_1435500	PLP2	perforin-like protein 2
exon_PVP01_1266300-E1	2292	195	8.508	1462.811	33.457	-1.756	0.640	-2.745	6.05E-03	4.85E-02	PVP01_1266300	AP3M	AP-3 complex subunit mu, putative
exon_PVP01_1439400-E1	789	30	3.802	653.751	5.427	2.405	0.876	2.745	6.05E-03	4.85E-02	PVP01_1439400		conserved Plasmodium protein, unknown function
exon_PVP01_0811100-E4	435	73	16.782	2885.372	13.190	2.091	0.762	2.744	6.08E-03	4.86E-02	PVP01_0811100		inner membrane complex sub-compartment protein 1, putative
exon_PVP01_1118100-E3	666	132	19.820	3407.751	17.733	-2.005	0.731	-2.743	6.09E-03	4.86E-02	PVP01_1118100	ERD2	ER lumen protein retaining receptor, putative
exon_PVP01_0722000-E2	834	40	4.796	824.636	6.869	2.297	0.838	2.741	6.12E-03	4.87E-02	PVP01_0722000		cyclin-dependent kinases regulatory subunit, putative
exon_PVP01_0807900-E1	1224	187	15.278	2626.808	25.967	-1.808	0.660	-2.740	6.15E-03	4.88E-02	PVP01_0807900		endonuclease, putative
exon_PVP01_1209400-E1	4752	170	3.577	615.093	28.355	1.770	0.646	2.740	6.14E-03	4.88E-02	PVP01_1209400		conserved Plasmodium protein, unknown function
exon_PVP01_0813400-E1	1920	300	15.625	2686.508	44.362	-1.475	0.539	-2.737	6.21E-03	4.92E-02	PVP01_0813400		conserved Plasmodium protein, unknown function
exon_PVP01_1212500-E1	816	205	25.123	4319.484	31.244	-1.614	0.590	-2.735	6.24E-03	4.94E-02	PVP01_1212500		tetratricopeptide repeat protein, putative
exon_PVP01_1243600-E2	1035	321	31.014	5332.524	54.330	1.606	0.588	2.734	6.27E-03	4.95E-02	PVP01_1243600	RFC3	replication factor C subunit 3, putative
exon_PVP01_1033100-E1	1146	87	7.592	1305.277	12.816	-2.071	0.758	-2.732	6.29E-03	4.96E-02	PVP01_1033100		conserved protein, unknown function
exon_PVP01_0916100-E1	1488	254	17.070	2934.938	34.181	-1.635	0.599	-2.728	6.37E-03	5.00E-02	PVP01_0916100	VP3	vivapain-3
exon_PVP01_1020100-E1	624	241	38.622	6640.497	33.880	-1.574	0.577	-2.729	6.36E-03	5.00E-02	PVP01_1020100	RAB1a	ras-related protein Rab-1A, putative
exon_PVP01_0212400-E1	510	279	54.706	9405.939	37.052	-1.753	0.643	-2.726	6.41E-03	5.02E-02	PVP01_0212400		conserved Plasmodium protein, unknown function
exon_PVP01_0602700-E1	393	60	15.267	2624.985	10.917	2.074	0.762	2.720	6.53E-03	5.11E-02	PVP01_0602700	ELC	myosin essential light chain ELC, putative
exon_PVP01_1205400-E2	738	71	9.621	1654.132	10.186	-2.245	0.826	-2.720	6.54E-03	5.11E-02	PVP01_1205400		cytidine and deoxycytidylate deaminase, putative
exon_PVP01_1414500-E1	6174	59	0.956	164.306	9.745	2.041	0.751	2.718	6.57E-03	5.13E-02	PVP01_1414500		conserved protein, unknown function
exon_PVP01_1459500-E2	1362	54	3.965	681.687	9.885	2.309	0.851	2.714	6.64E-03	5.17E-02	PVP01_1459500		GAS8-like protein, putative
exon_PVP01_0924800-E4	1137	225	19.789	3402.438	38.082	1.663	0.613	2.713	6.67E-03	5.18E-02	PVP01_0924800		thioredoxin-like protein, putative
exon_PVP01_1460400-E1	234	47	20.085	3453.426	8.258	2.106	0.778	2.708	6.76E-03	5.25E-02	PVP01_1460400		conserved Plasmodium protein, unknown function
exon_PVP01_1015000-E4	648	46	7.099	1220.537	7.855	2.184	0.809	2.700	6.94E-03	5.38E-02	PVP01_1015000		conserved protein, unknown function
exon_PVP01_0313400-E1	2922	111	3.799	653.147	19.400	1.739	0.645	2.698	6.99E-03	5.38E-02	PVP01_0313400		conserved Plasmodium protein, unknown function
exon_PVP01_0605200-E1	1542	136	8.820	1516.431	19.262	-1.980	0.734	-2.697	6.99E-03	5.38E-02	PVP01_0605200	RBM34	RNA-binding protein 34, putative
exon_PVP01_1329400-E1	636	83	13.050	2243.826	13.528	1.789	0.663	2.697	6.99E-03	5.38E-02	PVP01_1329400		conserved Plasmodium protein, unknown function
exon_PVP01_1116800-E1	2835	377	13.298	2286.422	61.509	1.659	0.616	2.696	7.02E-03	5.40E-02	PVP01_1116800	MCM6	DNA replication licensing factor MCM6, putative
exon_PVP01_0525400-E2	855	89	10.409	1789.749	15.463	1.758	0.652	2.695	7.04E-03	5.40E-02	PVP01_0525400		Plasmodium exported protein (PHIST), unknown function
exon_PVP01_1125200-E1	558	32	5.735	986.016	5.711	2.340	0.868	2.694	7.06E-03	5.41E-02	PVP01_1125200		CPW-WPC family protein
exon_PVP01_0214600-E1	1596	157	9.837	1691.356	23.862	-1.682	0.625	-2.693	7.08E-03	5.42E-02	PVP01_0214600		RNA-binding protein, putative
exon_PVP01_1251800-E1	537	32	5.959	1024.575	5.740	2.343	0.870	2.692	7.10E-03	5.42E-02	PVP01_1251800	PTP1	protein tyrosine phosphatase, putative
exon_PVP01_0823100-E1	762	258	33.858	5821.473	36.228	-1.555	0.578	-2.691	7.13E-03	5.43E-02	PVP01_0823100		proteasome subunit alpha type-3, putative

Exon ID	TL	Reads count	CVGE	RPKM	Mean counts	Log2FC	SD	Wald stat	p-value	q-value	Gene ID	Symbol	Product description
exon_PVP01_1332500-E3	1962	43	2.192	376.823	7.776	2.343	0.871	2.690	7.14E-03	5.43E-02	PVP01_1332500		conserved protein, unknown function
exon_PVP01_1452500-E1	1659	45	2.712	466.374	8.145	2.291	0.852	2.688	7.18E-03	5.46E-02	PVP01_1452500	MiGS	microgamete surface protein MiGS, putative
exon_PVP01_0820300-E1	894	422	47.204	8116.019	70.073	1.563	0.582	2.686	7.23E-03	5.48E-02	PVP01_0820300		conserved protein, unknown function
exon_PVP01_1324800-E2	888	610	68.694	11810.955	88.741	-1.362	0.507	-2.685	7.25E-03	5.49E-02	PVP01_1324800		60S ribosomal protein L5, putative
exon_PVP01_0923800-E1	2310	101	4.372	751.757	18.008	1.853	0.690	2.684	7.27E-03	5.49E-02	PVP01_0923800	LRR11	leucine-rich repeat protein
exon_PVP01_0934900-E1	1275	252	19.765	3398.275	35.154	-1.847	0.688	-2.683	7.29E-03	5.49E-02	PVP01_0934900	PDI-11	protein disulfide isomerase, putative
exon_PVP01_1118700-E5	1164	26	2.234	384.051	4.885	2.694	1.004	2.684	7.28E-03	5.49E-02	PVP01_1118700		TPH domain-containing protein, putative
exon_PVP01_0311300-E4	5223	124	2.374	408.197	21.538	2.072	0.772	2.682	7.31E-03	5.50E-02	PVP01_0311300		DNA polymerase alpha catalytic subunit A, putative
exon_PVP01_1313900-E1	5244	62	1.182	203.281	10.396	1.910	0.712	2.681	7.34E-03	5.51E-02	PVP01_1313900		conserved Plasmodium protein, unknown function
exon_PVP01_0205900-E1	804	404	50.249	8639.596	56.027	-1.797	0.671	-2.679	7.39E-03	5.52E-02	PVP01_0205900		cleavage and polyadenylation specificity factor subunit 5, putative
exon_PVP01_0311800-E1	3111	62	1.993	342.657	10.453	1.889	0.705	2.680	7.37E-03	5.52E-02	PVP01_0311800		RNA-binding protein, putative
exon_PVP01_0732500-E1	2016	680	33.730	5799.446	120.034	1.650	0.616	2.678	7.41E-03	5.52E-02	PVP01_0732500		tubulin delta chain, putative
exon_PVP01_0932200-E1	4533	64	1.412	242.752	9.932	-2.424	0.905	-2.678	7.40E-03	5.52E-02	PVP01_0932200		conserved Plasmodium protein, unknown function
exon_PVP01_1419900-E1	2334	94	4.027	692.461	16.660	1.873	0.699	2.678	7.40E-03	5.52E-02	PVP01_1419900		conserved Plasmodium protein, unknown function
exon_PVP01_0213100-E3	771	419	54.345	9343.891	61.429	-1.471	0.550	-2.675	7.46E-03	5.53E-02	PVP01_0213100		proteasome subunit alpha type-5, putative
exon_PVP01_0214900-E2	714	82	11.485	1974.621	12.913	-2.079	0.777	-2.676	7.45E-03	5.53E-02	PVP01_0214900	AOP	1-cys peroxiredoxin, putative
exon_PVP01_0421500-E1	1551	388	25.016	4301.184	62.011	1.393	0.521	2.675	7.47E-03	5.53E-02	PVP01_0421500		conserved Plasmodium protein, unknown function
exon_PVP01_0420800-E1	1893	80	4.226	726.620	13.974	1.980	0.741	2.673	7.52E-03	5.56E-02	PVP01_0420800		conserved Plasmodium protein, unknown function
exon_PVP01_0510500-E1	2076	278	13.391	2302.425	38.448	-1.535	0.575	-2.668	7.63E-03	5.62E-02	PVP01_0510500		DnaJ protein, putative
exon_PVP01_1019400-E1	651	54	8.295	1426.202	9.311	2.074	0.777	2.667	7.65E-03	5.62E-02	PVP01_1019400		conserved Plasmodium protein, unknown function
exon_PVP01_1118500-E1	870	312	35.862	6165.999	49.663	1.426	0.535	2.668	7.64E-03	5.62E-02	PVP01_1118500		tetratricopeptide repeat protein, putative
exon_PVP01_1219100-E3	1500	31	2.067	355.335	5.674	2.440	0.914	2.669	7.62E-03	5.62E-02	PVP01_1219100		DNA replication complex GINS protein, putative
exon_PVP01_0520900-E3	1218	420	34.483	5928.846	70.781	1.455	0.546	2.665	7.69E-03	5.62E-02	PVP01_0520900		conserved Plasmodium protein, unknown function
exon_PVP01_0944900-E2	816	41	5.025	863.897	7.412	2.253	0.845	2.665	7.70E-03	5.62E-02	PVP01_0944900		conserved Plasmodium protein, unknown function
exon_PVP01_1013600-E3	312	68	21.795	3747.334	9.232	-2.381	0.893	-2.665	7.69E-03	5.62E-02	PVP01_1013600		U6 snRNA-associated Sm-like protein LSm2, putative
exon_PVP01_1023700-E3	738	67	9.079	1560.941	11.651	1.963	0.737	2.665	7.70E-03	5.62E-02	PVP01_1023700	SIP	stripes inner membrane complex protein, putative
exon_PVP01_0942200-E1	894	38	4.251	730.826	6.660	2.243	0.842	2.663	7.75E-03	5.65E-02	PVP01_0942200		conserved Plasmodium protein, unknown function
exon_PVP01_1448000-E3	5619	52	0.925	159.115	9.382	2.260	0.849	2.662	7.76E-03	5.65E-02	PVP01_1448000		myosin J, putative
exon_PVP01_1119100-E1	1191	57	4.786	822.870	9.931	2.160	0.812	2.660	7.81E-03	5.67E-02	PVP01_1119100		conserved Plasmodium protein, unknown function
exon_PVP01_1249100-E1	1464	39	2.664	458.028	7.131	2.315	0.871	2.657	7.88E-03	5.71E-02	PVP01_1249100		conserved Plasmodium protein, unknown function
exon_PVP01_0112500-E3	1158	64	5.527	950.254	8.797	-2.342	0.882	-2.657	7.90E-03	5.71E-02	PVP01_0112500		tyrosine--tRNA ligase, putative
exon_PVP01_0809400-E1	1101	184	16.712	2873.417	26.838	-1.685	0.635	-2.655	7.92E-03	5.72E-02	PVP01_0809400	ZNF2	zinc finger protein, putative
exon_PVP01_1301000-E1	1296	28	2.160	371.468	5.270	2.586	0.974	2.656	7.91E-03	5.72E-02	PVP01_1301000		conserved Plasmodium protein, unknown function
exon_PVP01_0111000-E2	453	572	126.269	21710.307	84.702	-1.268	0.478	-2.654	7.96E-03	5.73E-02	PVP01_0111000		60S ribosomal protein L34, putative
exon_PVP01_0320200-E1	1893	71	3.751	1893	12.884	2.149	0.810	2.654	7.95E-03	5.73E-02	PVP01_0320200		conserved Plasmodium protein, unknown function
exon_PVP01_1457800-E1	2721	118	4.337	745.627	16.357	-1.926	0.726	-2.652	8.00E-03	5.74E-02	PVP01_1457800	FTSH1	ATP-dependent zinc metalloprotease FTSH 1, putative
exon_PVP01_0623100-E1	3663	184	5.023	863.672	26.079	-1.785	0.673	-2.651	8.02E-03	5.75E-02	PVP01_0623100		Plasmodium exported protein, unknown function
exon_PVP01_1429000-E1	6093	82	1.346	231.393	11.473	-2.314	0.873	-2.650	8.05E-03	5.76E-02	PVP01_1429000	CAF1	CCR4-associated factor 1, putative
exon_PVP01_0419900-E1	576	33	5.729	985.053	6.047	2.344	0.888	2.638	8.33E-03	5.94E-02	PVP01_0419900		MORN repeat protein, putative
exon_PVP01_0715900-E1	1977	580	29.337	5044.167	96.397	1.381	0.524	2.638	8.33E-03	5.94E-02	PVP01_0715900		conserved protein, unknown function
exon_PVP01_1139000-E3	954	99	10.377	1784.247	15.424	-1.969	0.746	-2.638	8.35E-03	5.95E-02	PVP01_1139000		50S ribosomal protein L19, putative

TL: transcript length; Coverage (CVGE) was calculated using the following equation: CVGE = (100* number of reads mapped to an exon) / Transcript Length (in bp); RPKM (reads per kilo base per million mapped reads was calculated using the following equation: RPKM = (number of reads mapped to an exon * 10^3 *10^6) / (total number of mapped reads from a given library * exon length (in bp)); Mean counts is the mean normalized counts, averaged over all samples from both conditions; Log2FC is the logarithm (to basis 2) of the Fold Change; SD: standard error estimate for the log2 fold change estimate; p-value: Wald test p-value ≤ 0,059 were considered; q-value: p-value adjusted for multiple testing with the Benjamini-Hochberg procedure which controls false discovery rate (FDR).
